# Tracking early mammalian organogenesis – prediction and validation of differentiation trajectories at whole organism scale

**DOI:** 10.1242/dev.201867

**Published:** 2024-01-31

**Authors:** Ivan Imaz-Rosshandler, Christina Rode, Carolina Guibentif, Luke T. G. Harland, Mai-Linh N. Ton, Parashar Dhapola, Daniel Keitley, Ricard Argelaguet, Fernando J. Calero-Nieto, Jennifer Nichols, John C. Marioni, Marella F. T. R. de Bruijn, Berthold Göttgens

**Affiliations:** 1 Department of Haematology, University of Cambridge, Cambridge CB2 0RE, UK; 2 Wellcome-Medical Research Council Cambridge Stem Cell Institute, University of Cambridge, Cambridge CB2 0AW, UK; 3 MRC Laboratory of Molecular Biology, Cambridge CB2 0QH, UK; 4 MRC Molecular Haematology Unit, MRC Weatherall Institute of Molecular Medicine, Radcliffe Department of Medicine, University of Oxford, Oxford OX3 9DS, UK; 5 Department of Microbiology and Immunology, University of Gothenburg, 405 30 Gothenburg, Sweden; 6 Department of Zoology, University of Cambridge, Cambridge CB2 3EJ, UK; 7 Wellcome Sanger Institute, Wellcome Genome Campus, Saffron Walden CB10 1SA, UK; 8 European Molecular Biology Laboratory, European Bioinformatics Institute, Saffron Walden CB10 1SA, UK; 9 Cancer Research UK Cambridge Institute, University of Cambridge, Cambridge CB2 0RE, UK; 10 Division of Molecular Hematology, Lund Stem Cell Center, Lund University, 221 00 Lund, Sweden; 11 Epigenetics Programme, Babraham Institute, Cambridge CB22 3AT, UK; 12 Altos Labs Cambridge Institute, Granta Park, Cambridge CB21 6GP, UK

**Keywords:** Cell fate and differentiation, Haematopoiesis, Mouse development, Single-cell transcriptomics

## Abstract

Early organogenesis represents a key step in animal development, during which pluripotent cells diversify to initiate organ formation. Here, we sampled 300,000 single-cell transcriptomes from mouse embryos between E8.5 and E9.5 in 6-h intervals and combined this new dataset with our previous atlas (E6.5-E8.5) to produce a densely sampled timecourse of >400,000 cells from early gastrulation to organogenesis. Computational lineage reconstruction identified complex waves of blood and endothelial development, including a new programme for somite-derived endothelium. We also dissected the E7.5 primitive streak into four adjacent regions, performed scRNA-seq and predicted cell fates computationally. Finally, we defined developmental state/fate relationships by combining orthotopic grafting, microscopic analysis and scRNA-seq to transcriptionally determine cell fates of grafted primitive streak regions after 24 h of *in vitro* embryo culture. Experimentally determined fate outcomes were in good agreement with computationally predicted fates, demonstrating how classical grafting experiments can be revisited to establish high-resolution cell state/fate relationships. Such interdisciplinary approaches will benefit future studies in developmental biology and guide the *in vitro* production of cells for organ regeneration and repair.

## Introduction

Single-cell transcriptomics has significantly contributed to our understanding of cell type diversity across species, organs and developmental processes. Efforts to build cell atlases from different model organisms include extensive transcriptomic profiling of embryonic development, with profiling of the mouse being particularly relevant given its broad use as a model for mammalian development. Independent efforts now provide coverage from embryonic days (E)3.5-E6.5, E6.5-E7.5, E4.5-E7.5, E6.5-E8.5, E6.5-E8.25, E9.5-E13.5 and E10.5-E15.0 (Argelaguet et al., 2019; Cao et al., 2019; Chan et al., 2019; Grosswendt et al., 2020; Han et al., 2018; He et al., 2020; Ibarra-Soria et al., 2018; Lescroart et al., 2018; Mittnenzweig et al., 2021; Mohammed et al., 2017; Pijuan-Sala et al., 2019; Scialdone et al., 2016), complemented by detailed analysis of specific organs such as the brain (La Manno et al., 2021) and the heart (de Soysa et al., 2019) or with emphasis on specific germ layers (Nowotschin et al., 2019). Combining datasets to provide an integrated timecourse over a longer timespan has been achieved by overcoming the challenge of integrating different sequencing technologies (Qiu et al., 2022). Of note, single-cell atlases of normal development have rapidly been used as a reference to interpret mutant phenotypes, providing new insights into the cellular and molecular processes controlled by key developmental regulators (Barile et al., 2021; Clark et al., 2022; Grosswendt et al., 2020; Guibentif et al., 2021; Mittnenzweig et al., 2021; Pijuan-Sala et al., 2019).

Integrating and annotating transcriptomic profiles in different contexts (e.g. across species, technologies, experiments, time points, etc.) represents a foundational step towards the construction and leveraging of cell atlases, but poses substantial challenges due to the difficulty of distinguishing between highly similar or transitional cell populations arising along complex differentiation trajectories. Without lineage tracing, computational inference of differentiation trajectories may provide useful information for understanding the dynamics of cellular diversification, but this needs to be interpreted with caution. The increasingly large number of methods for trajectory reconstruction furthermore highlights the need for benchmarking strategies (Saelens et al., 2019). Many trajectory inference methods are based on the concept of pseudotime, a latent dimension representing the transition between progenitors and differentiating cells as a function of transcriptional similarity. Though related, pseudotime and experimental time are different concepts. Thus, incorporating real time should improve reconstruction of developmental processes (Tritschler et al., 2019). Inspired by the Waddington landscape (Conrad Hal Waddington, 1957) and Optimal Transport theory (Monge, 1781), the probabilistic framework Waddington-OT (W-OT) takes advantage of experimental time and stochastic modelling to estimate the coupling probabilities of cells between consecutive time points and to reconstruct differentiation trajectories (Schiebinger et al., 2019).

Here, we report a densely sampled single-cell RNA-sequencing (scRNA-seq) atlas covering mouse development from E6.5 to E9.5 in 6 h intervals. This new atlas includes our 116,000 previously published E6.5-E8.5 transcriptomes that cover gastrulation and the initial phase of early organogenesis, complemented by 314,000 new E8.5-E9.5 transcriptomes that bridge a crucial gap in development not captured in existing datasets, i.e. the period of major morphological and organogenesis changes that occur between E8.5 and E9.5. This includes embryo turning, emergence of definitive-type haematopoietic cells, and initiation of the heartbeat and circulation. This new combined E6.5-E9.5 atlas delivers the most comprehensive transcriptome dataset for mammalian gastrulation and early organogenesis to date. To address the challenge of reconstructing and annotating cell lineages, we combined expert curation with a variety of computational methodologies to generate a resource of broad utility for the developmental biology community. We investigated further the intricate process of haemato-endothelial development, highlighting the multiple origins of endothelial cells and the formation of blood progenitors (BP) in asynchronous waves in spatially distinct sites. Moving beyond atlas generation, we also used precise embryo dissections to profile spatially defined areas of the developing embryo. Finally, computational cell fate predictions were contrasted with cell fates observed in state-of-the-art cell grafting experiments, where individual E7.5 primitive streak segments were orthotopically grafted into recipient embryos and resulting cell fates analysed after 24 h of culture by spatial and scRNA-seq analysis. Our study provides a blueprint for cell state/cell fate analysis during key stages of mammalian development, paving the way for future interdisciplinary studies of cell fates and origins.

## Results

### A densely sampled scRNA-seq atlas from E6.5 to E9.5 of mouse development

**Fig. 1:**
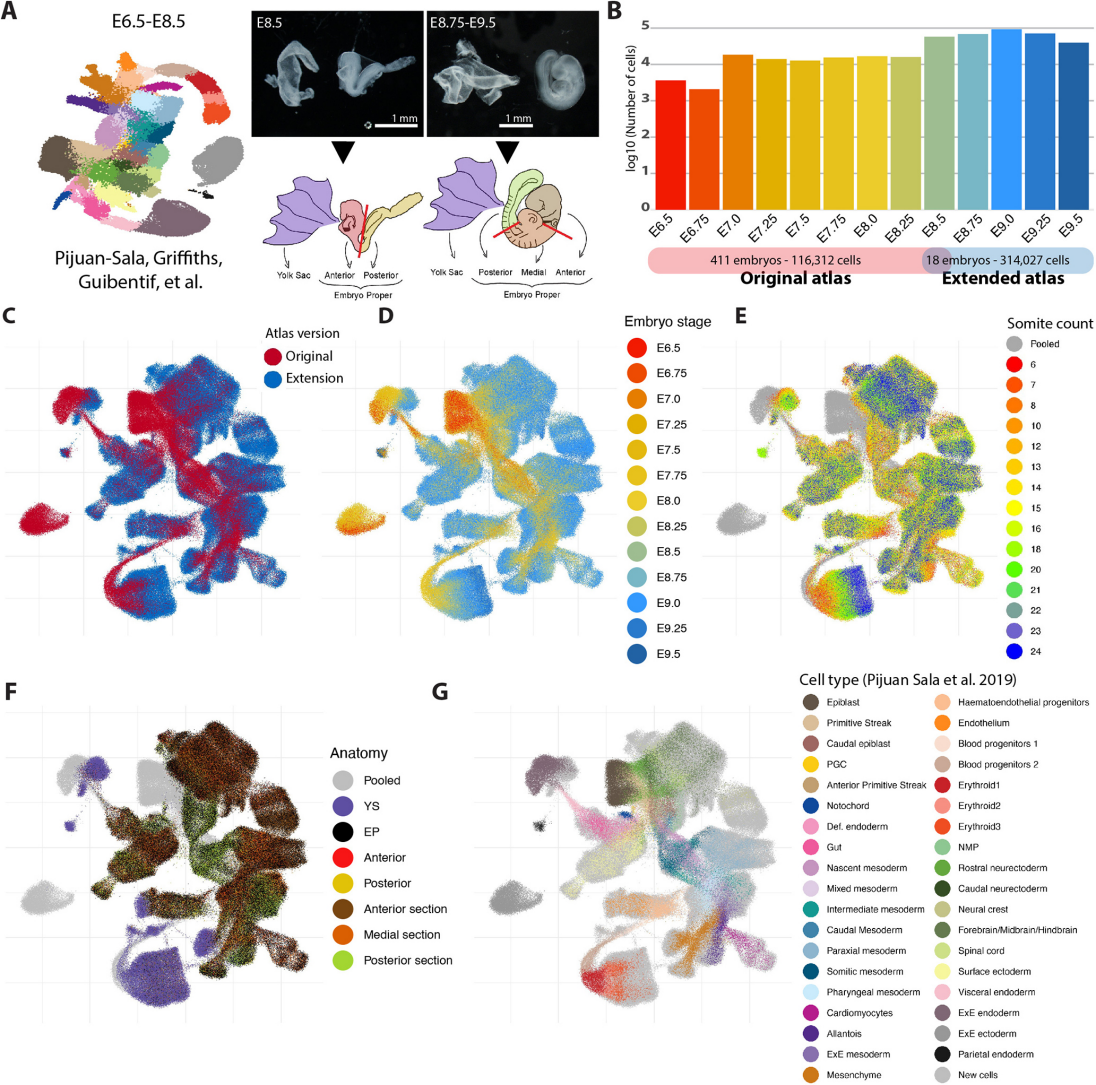
Extending a single-cell transcriptomic atlas of mouse gastrulation and early organogenesis. (A) Schematic of the experimental design. The publicly available E6.5-E8.5 timecourse experiment (Pijuan-Sala et al., 2019) was extended towards E9.5 across 24 h at each 6 h interval. An overlapping time point was generated (E8.5) to facilitate batch correction. Information of embryo dissections was recorded to support cell type annotations. (B) Bar plot showing the number of cells per time point after data integration. In total, the number of transcriptomic profiles increased from 116,312 to 314,027. (C-H) UMAP layout of the extended atlas. Cells are coloured by: (C) atlas version, the original atlas and the atlas extension; (D) time-point; (E) somite counts, as an alternative indication of developmental stage; (F) anatomical dissection (pooled cells correspond to the original atlas); (G) cell type annotations provided for the original atlas (newly generated profiles are highlighted in light grey). EP, embryo proper; ExE, extra-embryonic; NMP, neuromesodermal progenitor; PGC, primordial germ cell; YS, yolk sac.

We previously reported an scRNA-seq atlas covering mouse gastrulation and the early initiation of organogenesis between E6.5 and E8.5 (Pijuan-Sala et al., 2019). To capture the crucial organogenesis period between E8.5 and E9.5 we undertook a new timecourse experiment and integrated the new sampling time points (Fig. 1A). Thus, 116,312 cells distributed across nine time points from the original atlas were complemented with 314,027 new cells distributed across four new time points (E8.75-E9.5) as well as one overlapping time point (E8.5) to facilitate data integration (Fig. 1B). Combined, the new extended atlas, ranging from E6.5 to E9.5, contains 430,339 cells across 13 time points spanning 3 days of mouse development (Fig. 1A-C).

**Fig. 2:**
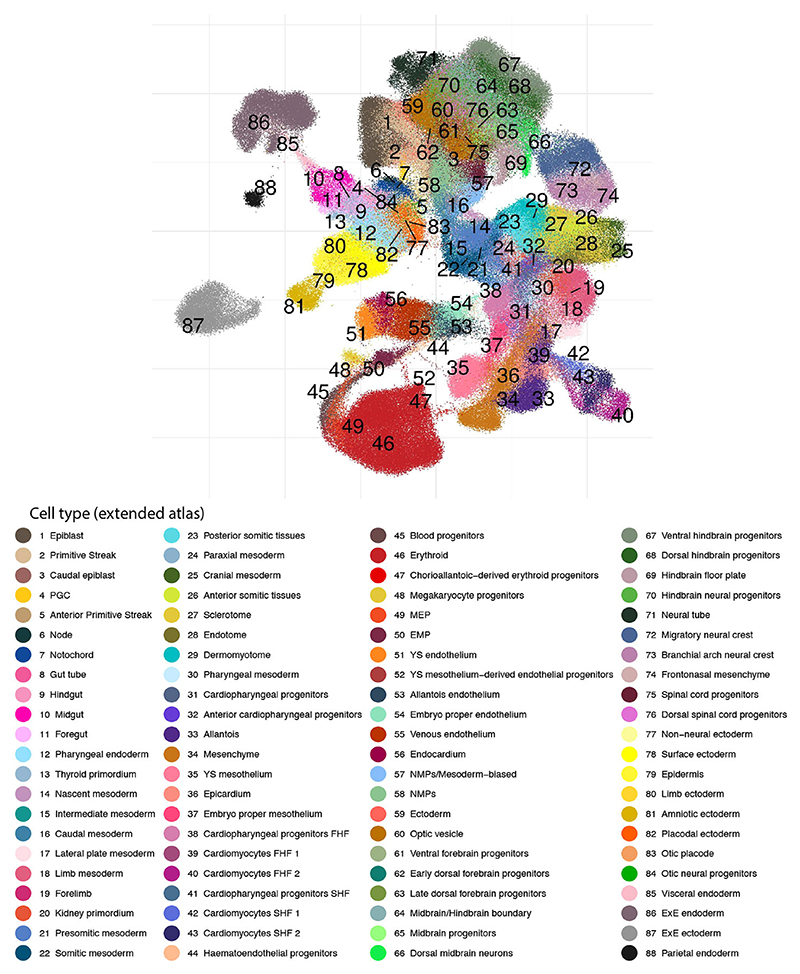
An extended transcriptional atlas of mouse gastrulation and early organogenesis. Cell type annotations resulting from the integration of both atlases and re-annotation process. EMP, erythroid-myeloid progenitors; ExE, extra-embryonic; FHF, first heart field; MEP, megakaryocyte-erythroid progenitors; NMP, neuromesodermal progenitor; PGC, primordial germ cell; SHF, second heart field; YS, yolk sac.

All embryos for the new dataset were dissected before droplet capture, to (1) profile yolk sac (YS) cells separately and (2) dissect the embryo proper to provide single-cell data anchored by anatomical location (Fig. 1A). As illustrated below, sequencing the various tissue segments independently aids the disambiguation of transcriptionally similar cell states (Fig. 1D-F). A combination of computational approaches and manual curation (see Materials and Methods, Fig. S1-S6 and Tables S1, S2) allowed us to define 88 major cell states, more than double the number identified in the previous atlas and reflecting the rapid diversification of cell states during the 24 h time window from E8.5 to E9.5 (Figs 1G and 2). The new combined dataset has been made freely available through a user-friendly web portal to be explored and leveraged by the wider scientific community (see Data availability).

### Inference of haemato-endothelial development reveals independent intra- and extra-embryonic trajectories

The computational reconstruction of developmental processes using single-cell transcriptomics remains a major challenge despite the large number of available methods. The timecourse experimental design of this atlas provides the advantage of incorporating developmental stage information, thus permitting the use of methods such as W-OT that anchor inferred developmental progression in real time rather than pseudotime (Schiebinger et al., 2019). In W-OT, developmental processes are modelled using a probabilistic framework that allows inferring ancestor and descendant probabilities between cells sampled at consecutive time points, thus allowing for time series analysis. We applied W-OT to the developing haematopoietic and endothelial systems, which are needed early during development to enable effective circulation once the heart starts beating at around E8.25-E8.5. Blood and endothelium arise in waves, at least some of which are thought to entail shared haemato-endothelial progenitors (reviewed by de Bruijn and Dzierzak, 2017; Elsaid et al., 2020). The first blood cells are so-called primitive erythrocytes, arising at E7.5 from mesodermal thickenings in the prospective blood islands of the YS. Next is a wave of definitive-type BPs that originate from the YS vasculature from E8.5. The haematopoietic stem cell (HSC) lineage is the last to emerge from the endothelium of major arteries of the embryo, the dorsal aorta and vitelline and umbilical arteries, starting from E9.5. Both YS definitive-type BPs and HSCs are derived from a specialised subset of endothelium, the haemogenic endothelium, through a so-called endothelial-to-haematopoietic transition. The extended mouse atlas covers the two YS-derived waves of blood emergence, as well as a variety of intra- and extra-embryonic populations of endothelial cells, providing a unique opportunity to explore the highly complex emergence and diversification of the initial haemato-endothelial landscape during mammalian development.

**Fig. 3:**
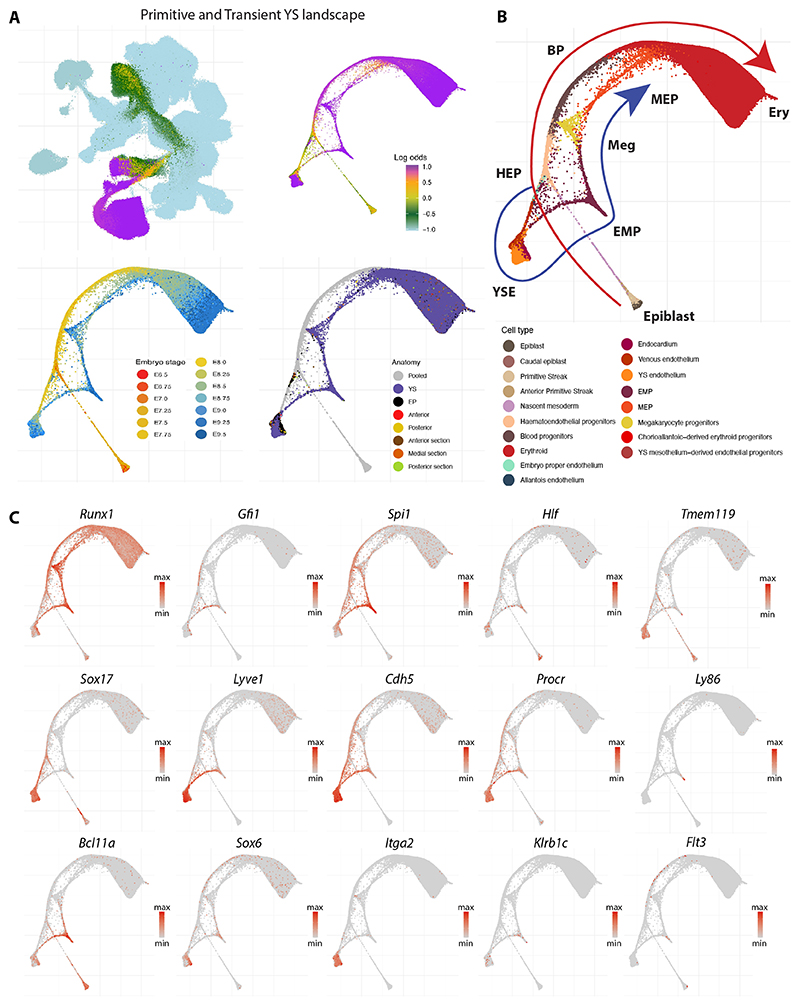
Primitive and definitive yolk sac waves of blood production. (A) UMAP layout of the mouse extended atlas displaying the log odds of fate probabilities associated with the primitive and yolk sac (YS) definitive haemato-endothelial landscape (top left). Cells with log odds >−0.5 were retained to generate a force-directed layout. Cells are coloured by log odds of fate probabilities of YS blood progenitors (BPs) and YS endothelial cells, embryo stage and anatomical region. (B) Force-directed layout with cells coloured by cell type, displaying the trajectories of the two distinct waves, clearly distinguished by time. (C) Force-directed layout showing a collection of gene markers associated with these populations, including those represented in small fractions as lymphocytes and microglial progenitors. EMP, erythroid-myeloid progenitors; EP, embryo proper; Ery, Erythroid; HEP, haemato-endothelial progenitors; Meg, megakaryocyte; MEP, megakaryocyte-erythroid progenitors; YSE, yolk sac endothelium.

To encompass all haemato-endothelial lineages, a W-OT fate matrix was computed for all blood and endothelial cell populations in the landscape [erythroid, megakaryocyte, megakaryocyte-erythroid progenitors (MEP), erythroid-myeloid progenitors (EMP), BPs, haemato-endothelial progenitors (HEP), endothelial populations from YS (YS EC), embryo proper (EP EC) and allantois (allantois EC), as well as venous endothelium and endocardium] leaving all other cells in the extended atlas grouped as ‘other fate’. In brief, a W-OT fate matrix is a transition probability matrix from all cells to several target cell sets at a given time point, commonly the end point of the timecourse experiment (here E9.25 and E9.5). Following this strategy, three main W-OT studies were performed. As a first proof-of-concept, only YS blood and endothelial cells were considered with E9.25-E9.5 cells from these two populations defined as targets (Fig. 3), which recovered the known divergence between the primitive and definitive-type YS waves over time. Specifically, the W-OT inferred primitive wave mainly generates nucleated primitive erythrocytes, as well as a small number of macrophage and megakaryocyte progenitors (red trajectory in Fig. 3B) (Tober et al., 2007). The second, definitive-type YS wave starts with the emergence of EMPs followed by MEPs (McGrath et al., 2015) arising from yolk sac haemogenic endothelium (YS HE) as highlighted by a collection of known markers for all these populations (blue trajectory in Fig. 3B, and gene marker inspection in Fig. 3C and Fig. S4-S6). To further characterise gene expression changes along the inferred primitive and definitive hematopoietic trajectories in the W-OT YS landscape, we employed Slingshot (Street et al., 2018) to infer lineage pseudotimes and used Tradeseq (Van den Berge et al., 2020) to identify gene changes associated with each trajectory (Fig. S7A,B and Table S3). It is worth noting that cells expressing lymphoid and microglial-like progenitor markers were also detected during the YS definitive wave, albeit at a low frequency (Fig. S8).

**Fig. 4:**
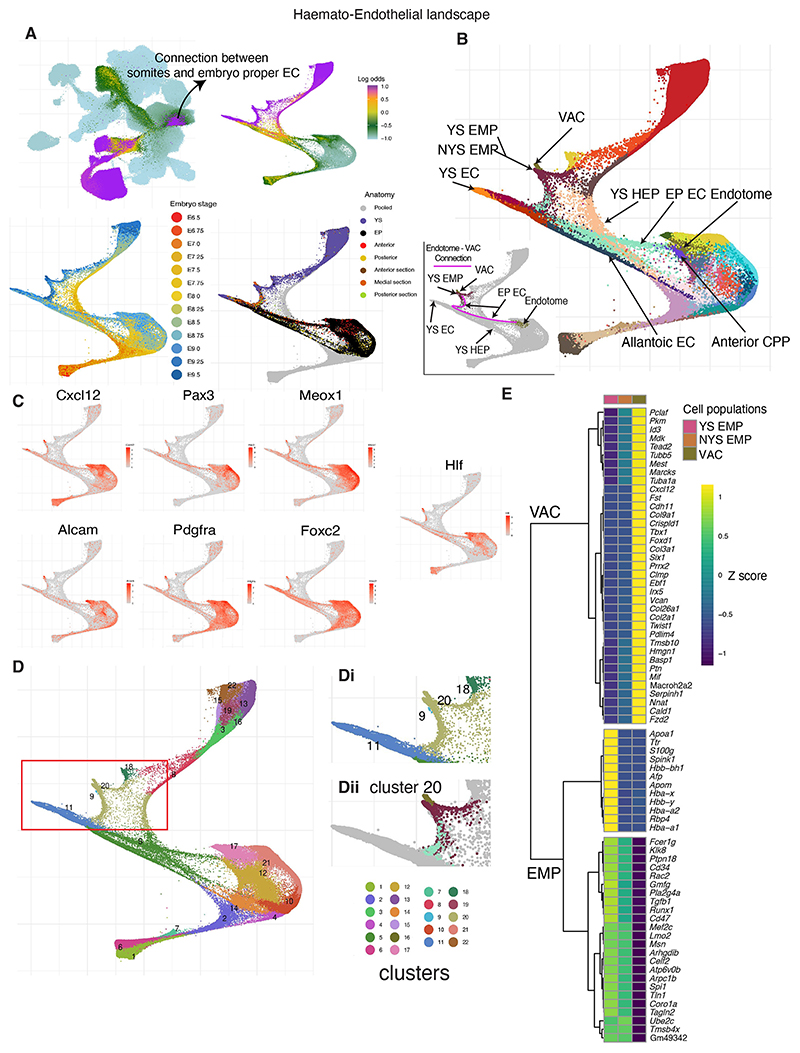
The haemato-endothelial landscape. (A) UMAP layout of the mouse extended atlas displaying the log odds of fate probabilities associated with a complete haemato-endothelial landscape (top left). Cells with log odds >−1 were retained to generate a force-directed layout. Cells are coloured by log odds of fate probabilities of all haemato-endothelial cells, embryo stage and anatomical regions. (B) Force-directed layout with cells coloured by cell type, highlighting multiple anatomical origins of haemato-endothelial cells and presence of blood cell types across yolk sac (YS) and embryo proper tissues. A subcluster of endotome cells is split from its major cell type origin and clustered together with EMPs (here named as VACs). Cells are coloured by cell type (see cell type labels at Fig. 1H). The bottom left plot highlights putative trajectories connecting embryonic endothelial cells, endotome cells and VACs in the landscape. (C) Collection of gene markers associated with the endotome population as reported by Nguyen et al. (2014) and Tani et al. (2020), and *hlf*, which has been associated with HSC induction (Yokomizo et al., 2019). Following the landscape in B, the purple line indicates the connection between embryonic endothelial cells, endotome cells and VACs in the landscape. (D) Newly calculated Louvain clusters identified in the landscape. The region of interest is highlighted by the red box and this region is displayed in Di. Cluster 20 is extracted and further split into anatomically distinct populations and used for differential expression and correlation analysis. Notice that cluster 9 are lymphocyte progenitors. (E) Heat map displaying differentially expressed genes across different cell populations clustered together in the region highlighted in Dii. CPP, cardiopharyngeal progenitor; EC, endothelial cell; EMP, erythroid-myeloid progenitors; EP, embryo proper; HEP, haemato-endothelial progenitor; NYS EMP: non-yolk sac EMP; VAC, vascular associated cells. Mean gene expression values were computed and scaled by rows (*z*-score).

For the second W-OT analysis, we wanted to explore the potential origins of endothelial cells together with blood cells, and thus considered the complete haemato-endothelial progenitor population, which resulted in a considerably more complex haemato-endothelial landscape as endothelial cells are found in multiple regions across the embryo (Fig. 4A,B, Fig. S9). For our third W-OT study, we merged blood cells with ‘other fates’ in the W-OT matrix, allowing us to focus explicitly on the complex endothelial landscape (third W-OT analysis). It is worth noting that, due to high levels of transcriptional convergence at endpoints during blood and endothelial differentiation, it is technically challenging to interrogate differentiation towards specific terminal cell types. Therefore, we opted to include multiple terminal blood and endothelial cell states in our W-OT analyses, allowing us to explore differentiation trajectories that produce molecularly convergent cell states from transcriptionally distinct starting populations. Importantly, the comprehensive metadata associated with our dataset makes it straightforward for the wider research community to explore alternative data analysis options that may be better aligned with their research questions.

Altogether, our W-OT landscapes revealed at least three putative endothelial differentiation trajectories that occur in different spatial locations and time windows during embryogenesis (Figs S10A and S11A-C). The first wave of endothelial production initiates early in the YS (Figs S7C and S11C). By contrast, the second wave(s) occurs in posterior regions of the embryo at intermediate time points (Fig. S11B,E) and the final wave initiates at later stages and occurs in anterior/medial sections (Fig. S11A,D). Dynamic gene expression changes were explored with Tradeseq (Van den Berge et al., 2020) along these inferred trajectories (Figs S7C and S11D-G) and detailed results of these analyses are presented in Tables S3-S5. In addition to revealing multiple spatiotemporally separated trajectories with different gene expression patterns, these analyses revealed a strong connection between somitic tissues and EP ECs, highlighted by differences in the UMAP coloured by log odds between the first landscape and the second/third landscapes (Fig. 3A versus Fig. 4A and Fig. S10A). This connection led to the identification of the endotome, a cell population not previously reported in mammalian embryos, which will be explored in the following section.

Finally, to identify distinctions between downstream endothelial populations we performed differential expression analysis (Fig. S10C). As expected, YS endothelium expressed *Lyve1*, venous endothelium showed high expression of genes associated with vasculogenesis and angiogenesis such as *Clec1b and Cldn5* and the endocardium expressed markers indicative of Bmp (*Id1* and *Id3*) and Notch signalling (*Hey1*). By contrast, embryo proper endothelium was relatively immature, as indicated by lower levels of endothelial genes such as *Cdh5, Pecam1* and *Dlk1*. Furthermore, embryo proper endothelial cells that arise in different anatomical locations expressed distinct sets of Hox genes (Fig. S10D).

### A previously unrecognised developmental trajectory involving intra-embryonic endotome-like cells

A small but clear subset of endothelium appeared to be part of a continuous differentiation trajectory originating from cells transcriptionally similar to endotome, a somitic subset defined in zebrafish embryos (Nguyen et al., 2014) (Fig. 4B and Fig. S11A,D). Added credence is given to this transcriptionally defined trajectory because both the contributing endotome and endothelial cells are derived from the same portions of the embryo (anterior/medial regions), demonstrating the utility of sub dissecting embryos before generating cell suspensions for sequencing. Furthermore, the mouse endotome cells are characterised by the expression of marker genes including *Cxcl12, Pax3, Meox1, Foxc2, Pdgfra* and *Alcam*, consistent with their recent discovery in zebrafish (Murayama et al., 2023; Nguyen et al., 2014; Tani et al., 2020) as well as by *Hlf* expression which has been associated with intra-aortic haematopoietic clusters (Yokomizo et al., 2019) (Fig. 4C). Further characterisation of the mouse endotome-like population (Fig. S12 and Tables S6 and S7) reveals it also expresses genes related to skeletal system development (*Snai1, Sox9, Tbx1, Tcf15, Twist1, Irx5, Pkdcc, Foxp1*), vasculature development (*Apoe, Nr2f2, Col3a1, Col4a1, Ednra*), muscle formation (*Fzd2, Six1, Tcf15, Twist1*) and VEGF signalling (*Vegfb, Vegfc*).

In the zebrafish embryo, endotome cells migrate towards the dorsal aorta and differentiate into endothelial cells that contribute to the niche for the emerging HSCs (Nguyen et al., 2014). Similarly, earlier studies on chick embryos showed a somite-derived cell population that contributes non-haemogenic endothelium, replenishing vascular cells that have undergone an endothelial-to-haematopoietic transition (EHT) (Pardanaud et al., 1996; Pouget et al., 2006; Sato et al., 2008). More recently, angioblast-like cells with similarities to somitic mesoderm-derived angioblast and endotome cells of chicken and zebrafish embryos were also reported in pluripotent stem cell-derived human axioloids (Yamanaka et al., 2023). In lower vertebrate models, the endotome was shown to originate at the ventral–posterior region of the sclerotome (Tani et al., 2020), which agrees with the transcriptional neighbours for the endotome population we discovered here in mouse embryos. It took our densely sampled and sub dissected scRNA-seq approach to discover a likely connection between the newly discovered mouse endotome and intra-embryonic endothelium (Fig. 4A-C, Figs S10A and S11A).

Intriguingly, in the Force Atlas representation of the haemato-endothelial landscape a subset of endotome-like cells present in later stage embryos (E9.25-E9.5) are placed next to EMP (Fig. 4D, red box). In addition, these cells cluster with EMPs (Fig. 4Di, cluster 20) when performing Louvain clustering using the top 50 principal components from the haemato-endothelial landscape. However, this association is only visually highlighted when a force-directed layout is generated on a subset of the atlas. By contrast, clustering, cell type annotation and UMAP embedding over the entire atlas, as well as the endothelial only landscape, demonstrates that these cells have a transcriptional identity aligned with earlier stage endotome cells that are transcriptionally distinctive compared with EMPs (Fig. 4Dii versus Fig. 2 and Fig. S11A).

Further exploration of these later stage endotome cells reveals that they lack a clear haemogenic signature (Fig. 4E, Fig. S13) and instead likely represent progenitors of endothelium and/or vascular mural and connective tissue cells, so-called vascular associated cells (VACs; Fig. 4B-E, Figs S11A and S13). Our densely sampled and regionally sub dissected atlas therefore suggests substantial transcriptional plasticity and developmental potential within mesodermal cells involved in the formation of intra-embryonic blood vessels. Future studies will be required to investigate how this plasticity extends to the specification of intra-embryonic HSCs within the vascular niche at E10.5.

### A gradient of heterogeneous molecular states along the anterior-posterior axis of the primitive streak

The complexity of this atlas is both an opportunity and a challenge for computational inference of developmental processes at the whole organism scale. Importantly, the atlas also provides a framework for complementary experiments which can in turn expand the impact of the atlas. Of particular interest to developmental biologists is the question of whether trajectory reconstruction analysis can unveil the timing at which cells start to differentiate towards either one or a set of specific cell fates. Although two-dimensional representations of single-cell expression data are often taken as a starting point, such representations lend themselves to overinterpretation, thus highlighting the need for experimental validation. We therefore complemented our extended atlas with (1) scRNA-seq of carefully dissected subregions of the primitive streak at E7.5 and (2) orthotopic transplantation of the same streak regions into recipient embryos followed by extended *in vitro* embryo culture and both microscopic as well as molecular fate analysis.

**Fig. 5:**
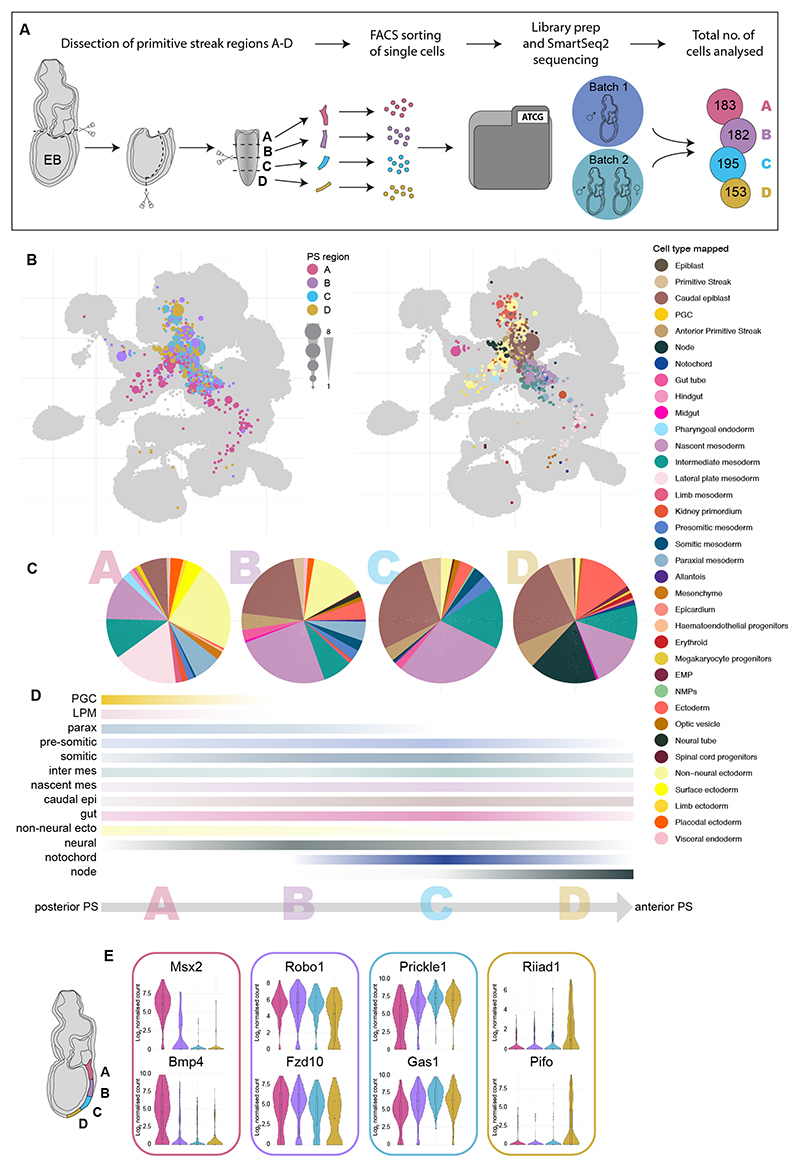
A gradient of transcriptomic differences between four sequential regions of the primitive streak at E7.5. (A) Schematic of the experimental set up. Single cells isolated from four sequential early bud (EB)-stage primitive streak regions were analysed by scRNA-seq. A total of three primitive streaks were analysed over two experiments. The final cell numbers analysed for each primitive streak region are indicated. (B) UMAPs of primitive streak cells mapped onto the extended mouse atlas. Cells are coloured by primitive streak region of origin (left) or transferred cell type label (right). The size of layout dots is proportional to the number of shared closest neighbours across primitive streak cells. (C) Pie charts showing the relative proportion of the different cell types of individual primitive streak cells mapped to by region of origin (A to D). Five cells from the most distal region D were unexpectedly annotated as erythroid progenitors (Fig. 5B,C). These may have inadvertently been included in the analyses during the removal of yolk sac tissues. (D) Percentile representation of cell type mapping along the primitive streak axis (from a to d). The colour intensity indicates cell type abundance and resembles the gradient of cell type bias from specific primitive streak portions. (E) Examples of the most differentially expressed genes between the four primitive streak regions. EMP, erythroid-myeloid progenitors; NMP, neuromesodermal progenitor; PGC, primordial germ cell.

Previous primitive streak cell transplant and labelling experiments produced a fate map of cells along the anterior-posterior axis of the gastrulating mouse embryo revealing reproducible region-specific contribution to all the major cell lineages of the embryo (Kinder et al., 1999). To link these data to the extended mouse atlas, we revisited these experiments and dissected the primitive streak of E7.5 (early bud; EB) mouse embryos into four sequential regions labelled A to D (with A being most posterior and D being most anterior), generated single-cell suspensions and profiled the regionally dissected cells using a modified Smart-seq2 scRNA-seq protocol providing deep coverage of each cell (Fig. 5A, Fig. S14). We mapped these primitive streak cells onto the extended atlas and employed label transfer to assign cell types (Materials and Methods). Approximately 27% of the isolated cells mapped to pluripotent cell types such as epiblast or primitive streak, 46% mapped to mesoderm, 24% to ectoderm and 3% to endoderm lineages (Fig. 5B).

There were clear differences across primitive streak regions (Fig. 5C,E). For example, only cells from the posterior-most region A mapped to lateral plate mesoderm (LPM) and primordial germ cells (PGCs) as well as expressing known posterior markers such as *Msx2* (Sun et al., 2016) (expressed in the allantois and known to be involved in PGC migration) and *Bmp4* (Fig. 5C-E, Fig. S15). In contrast, cells from the most anterior region D mapped to the node and expressed several brain and cilia-associated genes, including *Riiad1* and *Pifo* (also known as *Cimap3*) (Fig. 5E, Fig. S15). Cells from regions B and C were similar to each other and mapped to paraxial, presomitic and somitic mesoderm as well as neural and gut cells. However, these cell types were not unique to regions B and C and were found across the entire posterior-anterior axis of the primitive streak (Fig. 5C,D, Fig. S15). Consistently, *Robo1*, involved in central nervous system and heart development, *Fzd10*, associated with neural induction, *Prickle1*, a limb development gene, and *Gas1*, a somitic gene also present in LPM, all showed no bias for a particular segment and were expressed throughout the entire length of the primitive streak (Fig. 5E). Mesoderm-associated populations such as paraxial, presomitic and somitic mesoderm can be seen as transcriptionally defined subsets during later development (Guibentif et al., 2021), but these were not yet observed in this EB stage-derived primitive streak dataset. In summary, transcriptional signatures of cells obtained from anterior to posterior primitive streak regions were notably heterogeneous and already showed a bias towards particular molecular states.

### Cell fate analysis of orthotopic primitive streak grafts shows concordance between predicted and observed cell fates

**Fig. 6:**
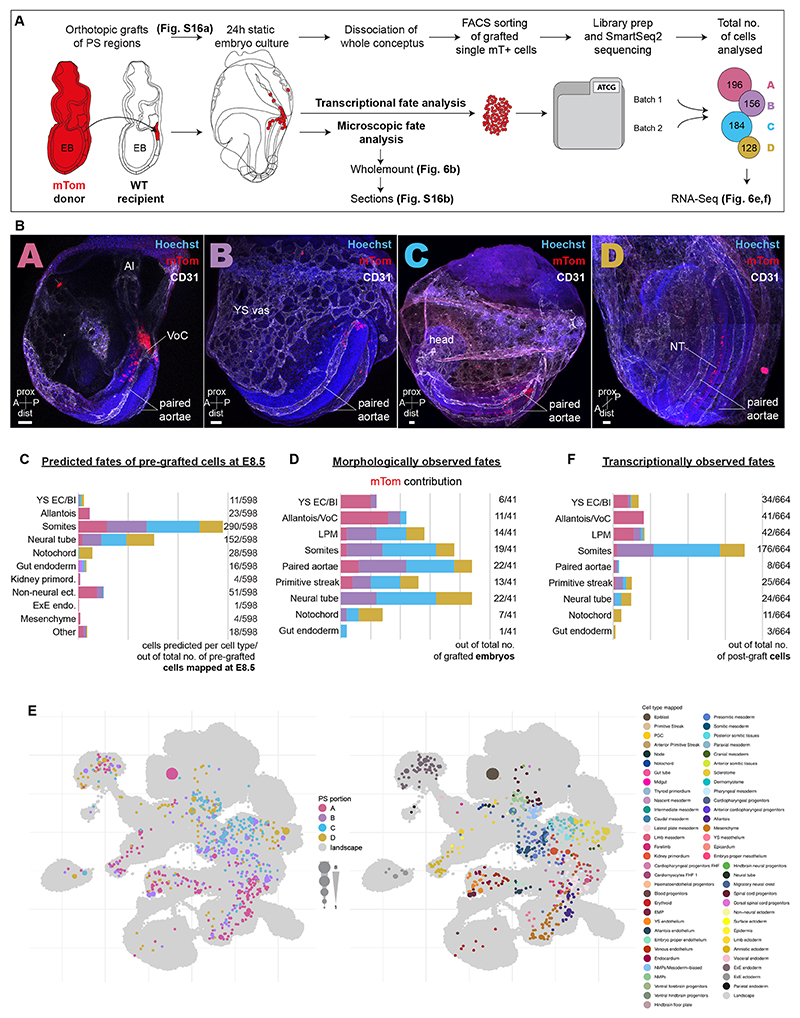
Experimental validation of cell fates in primitive streak grafts. (A) Schematic of the orthotopic graft experiments with subsequent analysis of cell progeny by imaging and RNA-seq. Primitive streak regions A to D isolated from E7.5 early bud (EB)-stage mouse embryos carrying a ubiquitous membrane-bound tdTomato (mTom) reporter were orthotopically grafted as small cell clusters into E7.5 (EB) wild-type recipient embryos. After 24 h of culture, grafted embryos were either fixed, stained for wholemount analysis and cryosectioned for detailed analysis of mTom^+^ cell contribution, or sorted for mTom^+^ single cells and processed for SmartSeq2 scRNA-seq. The total number of mTom^+^ cells sequenced is given. (B) Representative wholemount images of grafted embryos after culture, by region of origin (A to D), showing mTom (red) contribution to different tissues. Vasculature was stained with CD31 (white). Scale bars: 50 μm. (C) Fate map of predicted fates of cells freshly isolated from E7.5 (EB) primitive streak regions A to D (documented in Fig. 5), determined with the W-OT algorithm. Prediction was capped at E8.5 to allow direct comparison to microscopical and transcriptional fates of the post-grafted donor primitive streak-derived cells. (D) Fate map of experimentally observed fates of the primitive streak regions A to D based on microscopic analysis (wholemount and sections) of mTom contribution in different tissues of the grafted embryos after culture (Table S8). The number of grafted embryos with mTom contribution to particular tissues out of the total number of grafted embryos is shown for each fate. (E) UMAP of the mouse extended atlas highlighting the closest neighbouring cells to the transcriptomic profiles generated from mTom^+^ cells isolated from grafted embryos. Cells are coloured by primitive streak region (left) and transferred cell type label (right). The size of layout dots is proportional to the number of shared closest neighbours across primitive streak cells. (F) Fate map of transcriptionally observed fates based on the mapping of the post-grafted cells on the extended atlas. Only selected cell types are shown here to match the microscopically observed fates. The complete data are provided in Fig. S16D. A, anterior; Al, allantois; dist, distal; EC/BI, endothelial cell/blood islands; ecto., ectoderm; EMP, erythroid-myeloid progenitors; endo., endoderm; ExE, extra-embryonic; FHF, first heart field; LPM, lateral plate mesoderm; NMP, neuromesodermal progenitor; NT, neural tube; P, posterior; PGC, primordial germ cell; prox, proximal; VoC, vessel of confluence; YS vas, yolk sac vasculature.

Having identified molecular differences along the anterior to posterior axis of the primitive streak, we next used the extended cell atlas to predict the fates of regions A to D computationally and examined how these compare with experimentally determined cell fates. To this end, using computational fate inference, the closest neighbouring cells of the E7.5 primitive streak cells were identified in the atlas, and downstream fates were inferred based on the W-OT framework outlined above. Based on an initial survey of all predicted fates, a subset of major predictions was selected and visualised in so-called fate plots (Fig. 6C, Fig. S16E), which revealed associations between predicted cell fates and specific portions of the primitive streak. Cells from the posterior-most region A, for example, were primarily associated with mesodermal fates such as allantois, somites and the non-neural ectoderm, whereas the notochord and neural tube fates clearly favoured the most anterior region D (Fig. 6C, Fig. S16E).

To obtain experimental cell fate data, E7.5 embryos were orthotopically grafted with primitive streak regions A to D, cultured for 24 h and the fate contributions of the donor regions analysed at E8.25 by microscopic assessment and scRNA-seq (Fig. 6A). To facilitate analysis of cell fates, transgenic embryos carrying a ubiquitous membrane-bound tdTomato (mTom; Muzumdar et al., 2007) were used as graft donors. Cultured whole embryos and sections were immunostained (Fig. 6B, Fig. S16B) and careful observation of the location of mTom^+^ cells showed clear differences in tissue distribution between the donor primitive streak regions (summarised in Fig. 6D and detailed in Table S8). Notably, region A of the primitive streak contributed predominantly to the most posterior vasculature of the embryo, including the paired dorsal aortae and so-called vessel-of-confluence (VoC; Rodriguez et al., 2017), and to the allantois and some YS cells. Regions B and C also contributed to the paired dorsal aortae, as well as to somites, and to the LPM. Region D contributed mostly to the neural tube and notochord (Fig. 6D). To analyse the cellular contribution of the grafted primitive streak regions at the transcriptional level, we flow-sorted the mTom^+^ single cells from additional batches of cultured embryos and performed scRNA-seq (Smart-seq2). After low-level pre-processing, cells were mapped onto the extended atlas as before. Compared with the cells directly isolated from the E7.5 streak, donor primitive streak-derived cells in the E8.25 cultured embryos mapped to almost twice as many cell types (38 versus 63, respectively; compare Figs 5B and 6E), highlighting their advanced differentiation and diversification.

Comparison between the microscopically and transcriptionally-observed fates of the grafted primitive streak regions showed good concordance, with only subtle differences in the contribution to distinct lineages (Fig. 6D,F; microscopically observed fates are based on embryo counts, whereas transcriptionally observed fates are based on individual cell counts). Importantly, comparison between observed and predicted fates showed largely concordant patterns across computationally inferred fates, molecularly (scRNA-seq) mapped fates and microscopically assigned fates (Fig. 6C,D,F). It is worth noting that scRNA-seq-based analysis of fates has substantially enhanced granularity over microscopic analysis, with fate assignment based on our extended atlas for example providing up to 88 different cell types/molecular states for fine-grained annotation. In summary, combining multi-disciplinary approaches, we established primitive streak cell ‘end fates’ at a single-cell resolution. Placing these in the context of the extended mouse gastrulation and organogenesis atlas allowed establishing the proof-of-concept for a fate-predictive algorithm, able to forecast fate trajectories of individual primitive streak cells.

## Discussion

To realise the full potential of single-cell atlas efforts for developmental biology research, molecular profiling datasets need to (1) provide sufficient sampling density to enable a time-series capable of capturing rapid developmental processes and (2) contain enough single cells sequenced at reasonable depth, well annotated and provided to the broader community through user-friendly web portals. Here, we report such a resource covering the crucial stages of mouse gastrulation and early organogenesis, from E6.5 to E9.5 sampled every 6 h in 13 individual time steps. This dataset transforms our previous effort by more than tripling the number of cells and more than doubling the number of defined cell states. Furthermore, we revisit classical embryo grafting experiments with transgenic and single-cell analysis tools, providing a foundation for future efforts aiming to fully reconstruct cell lineage trees.

Previous single-cell atlas efforts from us and others placed observed molecular states into the context of existing knowledge of mouse development (reviewed by Tam and Ho, 2020) and further integrated by Qiu et al. (2022, 2023 preprint), altogether contributing to a deeper understanding of the molecular heterogeneity accompanying lineage differentiation during mouse embryogenesis. Our present study advances beyond these efforts in several impactful ways. By incorporating W-OT, spatial information and encompassing the developing YS region, our analysis achieves a comprehensive characterisation of blood and endothelial formation spanning the crucial developmental window of E8.5-E9.5. Our dense sampling, deep sequencing and regional sub dissection of embryos allowed us to identify cell states not previously observed in early mouse development, such as the endotome and VACs. Moreover, our trajectory analyses suggest that the endotome-like progenitor population is related to downstream endothelial cells, suggesting a greater intricacy in endothelial differentiation than previously appreciated.

Similar cell types have previously been described in zebrafish and chicken embryos and recently in pluripotent stem cell-derived human axioloids (Nguyen et al., 2014; Pardanaud et al., 1996; Pouget et al., 2006; Sato et al., 2008; Yamanaka et al., 2023), highlighting the value and complementarity of using a variety of different model organisms. Moreover, the zebrafish studies suggested that endotome and VACs may contribute to a cellular niche that promotes intra-embryonic formation of blood stem/progenitor cells (Nguyen et al., 2014). In the mouse, the best-understood site of HSC emergence is the haemogenic endothelium of the dorsal aorta where pro-HSCs are generated from E9.5 (Rybtsov et al., 2014). These haemogenic endothelial cells are lateral plate- or splanchnopleuric mesoderm-derived and were shown to be replaced by somite-derived endothelial cells concomitant with the extinction of HSC generation (Pouget et al., 2006). Interestingly, another report suggests that 7 days later during mouse gestation, endothelial cells in fetal bone marrow undergo haemogenic transdifferentiation and produce BPs and differentiated cells (Yvernogeau et al., 2019). Pax3-Cre lineage tracing further suggested a somitic origin of those haemogenic cells.

Intriguingly, more recent unpublished Tbx6^+^ (Yvernogeau et al., 2020 preprint) and Pax3^+^ lineage tracing studies (Lupu et al., 2022 preprint) have been performed at embryonic time points (E7.5-E11.5) exploring developing anatomical regions (anterior/medial embryonic sections) that overlap with the emergence of endotome, VACs and endotome-derived endothelial cells in our extended atlas. These studies suggest that somitic mesodermal precursors give rise to endothelial cells of the limb and trunk region in developing mouse embryos, as well as stromal cells juxtaposed to the dorsal aorta, which may be like the VAC population we identify in this present study. In these studies, somite-derived endothelial cells make only a minimal contribution to dorsal aorta endothelium by E9.5-10.5. Future work will need to dissect the underlying connection we identify here between a putative endotome cell population, VACs and embryo proper endothelium. Such studies will: (1) enhance our understanding of early blood and endothelium development, (2) likely reveal principles relevant to cell plasticity and potential in other developmental contexts, and (3) provide new mechanistic insights that could be exploited to control differentiation, for example for directed differentiation of pluripotent cells for cell therapy applications.

By combining scRNA-seq with classical embryo grafting experiments, we show how single-cell atlases provide a powerful resource to revisit classical concepts of developmental biology. The results presented in this study are limited in scope but already illustrate how timecourse-guided trajectory reconstruction performed with computational methods such as W-OT represents a promising approach to dissect complex developmental processes. Our findings also support substantial fate bias present in the E7.5 primitive streak, based on deeply sequenced single-cell profiles. As we did not perform heterotopic transplants, our experiments could not assess fate plasticity nor full fate potential. Moreover, deeper and more comprehensive transplant experiments should be designed around new experimental tools not available when this work was performed, including extended *in vitro* embryo culture (Aguilera-Castrejon et al., 2021), as well as single-cell barcoding to permit reconstruction of single-cell phylogenies (Bowling et al., 2020).

A future thereby emerges where a confluence of complementary technologies will transform our understanding of early mouse development to a level previously only attained with non-mammalian organisms. Building on data resources such as the one reported here, mechanistic insights will continue to require perturbation experiments, with the important proviso that carefully chosen experimental perturbations have the added benefit to provide new insights into disease processes, in particular congenital defects associated with mutations in developmental regulator genes. Our extended mouse gastrulation atlas provides the developmental biology community with a significant new resource to probe novel hypotheses concerning cell fate acquisition and lineage commitment during embryogenesis.

## Materials and Methods

### E8.5-E9.5 embryo collection for the extended atlas

All procedures were performed in strict accordance to the UK Home Office regulations for animal research. Mouse embryos were collected under the project licence number PPL 70/8406. Animals used in this study were 6-10 week-old females, maintained on a lighting regime of 14 h light and 10 h dark with food and water supplied *ad libitum*. Following wild-type C57BL/6 matings, females were killed by cervical dislocation at E8.5, E8.75, E9.0, E9.25 and E9.5. The uteri were collected into phosphate buffered saline (PBS) with 2% heat-inactivated fetal calf serum (FCS) on ice and the embryos were immediately dissected and processed for scRNA-seq. For each time point, four embryos were selected based on morphology and somite counts, in order to span the range expected for the given time point according to Davidson (1989) and processed individually. The exception is the E9.5 time point, where embryos were smaller than expected and only two embryos were collected with lower somite numbers. The YS was systematically separated from the rest of the embryo and processed as a separate sample. Of the four selected embryos at each time point, two were partitioned in defined anterior-posterior sections dissociated as separate samples and two were dissociated as bulk and the suspension then divided into two separate samples for 10x RNA-seq analysis. For the E8.5 partitioned embryos, they were divided into two halves, with the cut being made at the fourth somite level (i.e. right before the fourth somite level). The anterior portion (including headfolds, branchial arches and heart rudiment) and the posterior portion (including allantois, hindgut, primitive streak) were processed as individual samples. For E8.5 bulk-dissociated embryos, the single-cell suspension was divided into two separate samples for 10x RNA-seq analysis.

E8.75-E9.5 embryos were divided into three segments, with cuts made below the otic pit and below the heart (at the 10th-12th somite level). The anterior-most portion (including brain structures anterior to rhombomere 6 and branchial bars), mid-portion (including the heart and remains of vitelline vessels) and posterior portion (including allantoic structures, hindgut and posterior-most somites) were then singularised and further processed as separate samples. Single-cell suspensions were prepared by incubating the samples with TrypLE Express dissociation reagent (Life Technologies) at 37°C for 7 min under agitation and quenching in PBS with 10% heat-inactivated serum. The resulting single-cell suspension was washed and resuspended in PBS with 0.4% bovine serum albumin and filtered through a Flowmi Tip Strainer with 40 μm porosity (Thermo Fisher Scientific, 136800040). Cell counts were then assessed with a haemocytometer. Single-cell RNA-seq libraries were generated using the 10x Genomics Chromium system (version 3 chemistry), and samples were sequenced according to the manufacturer’s instructions on the Illumina NovaSeq 6000 platform.

### Publicly available mouse gastrulation data

The mouse gastrulation atlas was processed exactly as described in Pijuan-Sala et al. (2019). This timecourse experiment contains 116,312 cells distributed across nine time points sampled across E6.5-E8.5 at 6 h intervals. Only two samples of this dataset (where a sample is a single lane of a 10x Chromium chip) contained pooled embryos staged across several time points. Cells from these samples are denoted as ‘mixed gastrulation’ in the metadata.

### 10x Genomics data low level analysis

Raw reads were processed with Cell Ranger 3.1.0 using the mouse reference 1.2.0, mm10 (Ensembl 92) and default mapping arguments. The following steps of pre-processing were performed with R using the same functions, parameters and software versions broadly described in Pijuan-Sala et al. (2019): swapped molecule removal, cell calling, quality control, normalisation, selection of highly variable genes, doublet removal, batch correction and stripped nucleus removal. Therefore, singularity containers available in https://github.com/MarioniLab/EmbryoTimecourse2018 provide the necessary software for reproducing these steps.

### Generating an integrated atlas

Log transformed normalised counts obtained from Pijuan-Sala et al. (2019) and those generated here were integrated into an extended timecourse experiment across mouse developmental stages E6.5-E9.5 (13 time points). Highly variable genes (HVGs) were calculated using ‘trendVar’ and ‘decomposeVar’ from the scran R package, with loess span of 0.05. Genes that had significantly higher variance than the fitted trend (Benjamini–Hochberg-corrected *P*<0.05) were retained. Genes with mean log2 normalised count <10^–3^, genes on the Y chromosome, the gene *Xist* and the reads mapping to the tdTomato construct (where applicable) were excluded. Hence, this expression matrix contained 23,972 genes and 430,339 cells. Batch correction with fastMNN function from scran (Lun et al., 2016) was performed as described above, resulting in 5665 genes and 75 batch-corrected principal components.

### Mapping stage and cell type annotations within the extended gastrulation atlas

Metadata annotations such as embryonic developmental stages and cell types were assigned to the mixed time points (annotated as mixed gastrulation in the metadata) and newly generated E8.5 samples, respectively, using a strategy based on fastMNN. In this approach, UMI counts from both the reference and the query datasets are merged, normalised and log transformed together. Then, HVGs and top principal components are computed to subsequently use fastMNN for re-scaling the principal component analysis (PCA) space from both datasets. The annotations from the reference metadata are assigned to the query data as the mode among k nearest neighbours (KNN) between the query and the reference PCA subspaces using queryX function from Biocneighbours. The number of nearest neighbours is chosen depending on the resolution of transferred annotations. Mixed gastrulation time points were allocated to embryonic developmental stages using the 30 nearest neighbours queried from the top 50 batch-corrected principal components from the E6.5-E8.5 reference dataset. New E8.5 cell type annotations were assigned using ten nearest neighbours with respect to E8.5 cells from the reference atlas in the corresponding E8.5 subspace of the integrated batch corrected PCA described above.

### Constructing optimal transport maps

The W-OT approach was conceived to model timecourse experiments of developmental processes as a generalisation of a stochastic process using unbalanced optimal transport theory. Thus, it allows estimating the coupling probabilities between cells of consecutive time points, while taking into account cell growth and death (Schiebinger et al., 2019). The transport maps of consecutive time points were constructed over the entire set of cells with W-OT (1.0.8.post1) using default settings, except for skipping the dimension-reduction step, and instead using the batch-corrected principal components as input as well as three iterations for learning the cell growth rate. Embryonic stages E9.25 and E9.5 were collapsed into a single time point for computing the transport maps because the somite count (a more accurate measure of developmental stage) overlapped between these embryos.

### Estimating the descendants from cell populations at E8.5

The transport maps explained above were used to estimate the full trajectories of every cell population present at E8.5. That is, for each cell population (i.e. Erythroid3) the coupling probabilities were used to reconstruct the sequences of ancestor and descendant distributions by pushing the cell set through the transport matrix backwards and forwards, respectively. Cells were allocated as descendants from a cell population at E8.5 by selecting those with maximum mass across all trajectories at time points E8.75-E9.5.

### Integrating the brain and gut development atlases to support cell type annotations

Mapping publicly available data from the atlas of brain development (La Manno et al., 2021) and both atlases of gut development (Nowotschin et al., 2019) against the extended gastrulation atlas was performed following the strategy based on fastMNN mentioned above. However, in this case the matrices of principal components from the query dataset were randomly split into subsets smaller than 10,000 cells for fastMNN and merged back to generate a single mapping output.

### Expansion and refinement of cell population annotations

A combination of complementary strategies was used for defining final cell type annotations (Fig. S1). First, cell type annotations from Pijuan-Sala et al. (2019) were transferred within overlapping time points (E8.5) and cell descendants were estimated as described above (Fig. S1A,B). The landscape was then split into two subsets, a mesodermal and an ectodermal-endodermal landscape (the latter including neuromesodermal progenitors) (Fig. S1C), and subsequently clustered using the scanpy implementation of the Leiden algorithm (Traag et al., 2019) with a resolution of 5 (Fig. S1D). Highly variable genes and batch corrected principal components were recomputed on the subsets before clustering. Then, annotations from the original atlas expanded through mapping and estimation of cell descendants were manually refined by means of differential expression analysis between clusters (using findMarkers from the scran package version (Lun et al., 2016), as well as literature and visual inspection of gene markers resulting in major 88 cell type populations (Fig. 2). In addition, we used the FindAllMarkers (min_pct=0.25, logfc_threshold=0.25) function in Seurat (4.2.0) (Hao et al., 2021) to identify marker genes for all 88 cell types (Table S1).

### Generating the landscapes of haemato-endothelial trajectories

The strategy for generating the haemato-endothelial landscapes was based on the so-called W-OT fate matrix. In brief, a W-OT fate matrix is a transition probability matrix (the rows of this matrix add up to 1) from all cells to a number of target cell sets at a given time point, commonly the ending point of the timecourse experiment. To fully cover the haemato-endothelium, three W-OT fates matrices were computed, one for each landscape presented in our results. One using E9.25-E9.5 YS blood and YS endothelial cells as targets, another one with YS blood as well as both YS and embryonic endothelial tissues, and a third one where only endothelial cell types were considered targets (Figs 3, 4 and Fig. S10, respectively). Then, to identify cells that form a differentiation trajectory towards any ofthe abovementioned cell fates, for every cell in the landscape the likelihood probabilities associated with these fates and the remaining cells at E9.25-E9.5 cell types (i.e. cells grouped as ‘other fate’) were compared using the log odds or ratio of probabilities. The log odds is defined as the logarithm of the ratio of probabilities of two different categorical and mutually exclusive outcomes log(*p*/1−*p*). These ratios provided a quantitative setting for more interpretable thresholds when selecting cells that potentially belong to a cell fate trajectory. These cells are then retained and used to generate new layouts including only potentially fated cells towards blood and endothelium: that is, selecting pluripotent and mesodermal cells above a reasonable log odds threshold, recomputing HVGs, correcting for batch effects and generating new force-directed graphs from inferred trajectories. Cells with log odds >0 were considered fate biased towards the set of selected population targets, such as in the proof of concept performed in Fig. 3. As the log odds is computed by summing over the cell probabilities for all population targets (here denoted as *p*), divided by the probability of all other fates grouped together (1-*p*); cells with negative values but close to 0, although with higher uncertainty, might well be contributing to the population targets of interest. Thus, for more complex landscapes the threshold was lowered to log odds >−1 (Fig. 4, Fig. S10) in order to exclude all cells not associated with haemato-endothelial fates with high confidence, while keeping a higher degree of uncertainty for those retained.

### Visualisation

To generate the UMAP layout of the whole embryo, we used the top 50 batch corrected principal components to generate a BBKNN graph (Polanski et al., 2020) and then the scanpy implementation of UMAP, with parameter min_distance=0.99. To generate the force-directed layouts of the haemato-endothelial landscapes, we recomputed HVGs for each subset as well as batch correction of PCA manifolds (we again retained the top 50 principal components). We then built a KNN graph (K=50) and used ForceAtlas2 (Jacomy et al., 2014) implementation of force-directed layouts included in scanpy.

### Slingshot trajectory inference

Slingshot (2.7.0) (Street et al., 2018) was used, in a semi-supervised fashion, to infer differentiation trajectories and produce average pseudotimes along various putative lineage trajectories that were highlighted during W-OT when generating the YS landscape (Fig. 3 and Fig. S7; primitive, YS definitive and YS endothelial trajectories) and the endothelial landscape (Figs S10 and S11; anterior/medial and posterior trajectories) using the getLineages and getCurves functions (default settings, starting clusters were provided). The first 45 batch corrected principal components were provided to getLineages as input data for the respective landscapes as well as cluster labels generated using the default Seurat (4.2.0; Hao et al., 2021) functions FindNeighbors (dims=1:50, reduction=‘PCA’) and FindClusters (resolution=3) for the respective landscapes. Cells that were members of relatively small clusters (<100 cells) were excluded by setting their cluster value equal to −1 when running getLineages. Before running getLineages, supervised filtering of cells based on metadata (stage, anatomy and predicted anatomy labels) was performed. Predicted anatomy labels for embryo proper cells in the haemato-endothelial landscape were determined by performing label transfer from neighbouring cells with specific anatomical labels (anterior, posterior, anterior section, medial section and posterior section) to cells from embryo proper sections using the embeddingKNN function from StabMAP (Ghazanfar et al., 2023) (type=’uniform_fixed’, k_values=5, cords=top 50 PCs). For the YS primitive blood trajectory inference, cells from stages E6.5-E8.25 were considered. For the YS definitive blood trajectory, cells from stages E7.75-E9.5 were considered. Finally, for the YS endothelial trajectory inference cells from the YS and pooled regions were considered and blood cells (EMP, MEP, BPs, erythroid, megakaryocyte progenitors) were excluded. For the anterior/medial endothelial trajectory inference, cells from stages E8.5-E9.5 were considered and cells from anatomical posterior section and posterior were excluded, as well as embryo proper cells with predicted anatomy labels of posterior section and posterior. For the posterior endothelial trajectory inference, cells from stages E7.5-E9.5 were considered and cells from anatomical anterior section, medial section and anterior were excluded, as well as embryo proper cells with the same predicted anatomy labels (anterior section, medial section and anterior). When multiple, similar lineages towards downstream blood or endothelial cells were predicted by getLineages, average pseudotimes were calculated using the slingAvgPseudotime function. These average pseudotimes were used in subsequent Tradseq analyses.

### Tradeseq differential gene expression analyses

To identify genes with altered expression over the inferred trajectories the average pseudotimes were provided to Tradeseq (1.10.0; Van den Berge et al., 2020). Generalised additive models were fit (fitGAM, nknots=6, cellWeights=1) to genes that were highly variable [top 2000 highly variable genes were identified using VariableFeatures function in Seurat (4.2.0)] among the cells that were part of the inferred trajectories. The associationTest function was then used to identify which of these genes had altered expression over the inferred trajectories. ComplexHeatmap (2.15.3; Gu et al., 2016) was used to visualise the expression patterns of the genes with the highest 300 waldStat scores (*P*-value<0.01 and meanLogFC>2). Metascape (Zhou et al., 2019) was used to identify Gene Ontology (GO) terms that were enriched for clusters of genes that were associated with the anterior/medial and posterior endothelial differentiation trajectories (Tables S4 and S5).

### Cell communication analyses with CellChat

To identify predicted ligand-receptor interactions among cells that were present in the YS landscape, the haemato-endothelial landscape and the anterior/medial sections of the cells in the haemato-endothelial landscape (Table S7), we used the computeCommunProb (default settings) and filterCommunication (min_cells=10) in CellChat (1.6.1; Jin et al., 2021).

### Differential gene expression and canonical correlation analysis of endotome-derived VACs

Subsequent to the construction of a complete haemato-endothelial landscape, Louvain (Subelj and Bajec, 2011) clusters (as implemented in the R package igraph; https://igraph.org) were generated from the batch-corrected principal components obtained as explained above (Fig. 4C). Cluster 20 was of particular interest for investigating both shared and non-shared gene expression profiles between the anatomically distinct populations YS EMP and non-yolk sac (NYS) EMP, and a subset of endotome cells that is placed next to them (Fig. 4D,E). Differential gene expression analysis was performed using the function findMarkers from the scran package (Lun et al., 2016). The statistical test performed by findMarkers uses a general linear model and moderated t-statistics to perform differential expression, as implemented in the R package limma (Ritchie et al., 2015). The threshold established was FDR<0.05 and LogFoldChange>0.5. Furthermore, only genes among the top 20 rank genes are displayed in heatmaps (considering that some genes can have tied ranks, gene lists usually contain more than 20 genes). CCA as implemented in the R package CCA (Gittins, 1985; Kanti Mardia and Bibby, 1979) was applied to mean metacell expression values of YS EMPs and the endotome-derived VACs. Positively correlated genes were then identified by selecting only those with positive coefficients (canonical scores) over the two canonical variates and visualised using a scatter plot fitting a linear regression. Importantly, such analysis was performed using Metacells (Baran et al., 2019) to strengthen gene expression signals.

### Metacells

We identified metacells (i.e. groups of cells that represent singular cell states from single-cell data) with the goal of achieving a resolution that retains the continuous nature of differentiation trajectories while overcoming the sparsity issues of single-cell data. We adopted the SEACells implementation (Persad et al., 2023). Following the method guidelines, metacells were computed separately for each sample using approximately one metacell for every seventy-five cells. Following metacell identification, we regenerated the gene expression matrices summarised at the metacell level. Sample-specific count matrices were then concatenated and normalised together. Metacells were used exclusively to inspect the correlation between genes expressed in YS EMPs and endotome-derived VACs, as well as the lack of a clear haemogenic signature. However, metacells were computed for all the haemato-endothelial landscapes.

### Smart-seq2 data low level analysis

Sequencing reads were aligned against the mm10 genome following the GRCm38.95 genome annotation (Ensembl 95) using STAR version 2.5 (Dobin et al., 2013) with –intronMotif and 2-pass option mapping for each sample separately (i.e. –twopassMode basic) to improve detection of spliced reads mapping to novel junctions. Sequence alignment map (SAM) files were then processed with samtools-1.6 in order to generate the corresponding count matrix with htseq-count from HTSeq, version 0.12.4. Cells with total counts lower than 10,000 reads were filtered out from downstream analysis based on observed library size distributions. The mitochondrial fraction of reads per cell and library complexity were computed as part of the quality control (QC). Only two cells showed a mitochondrial fraction larger than 4% and the lowest number of genes detected was 3113. Thus, no cells were excluded during QC. Size factors were computed with scran and log-transformed normalised counts were obtained using the logNormCounts function from scater using size factors centred at unity before calculation of normalised expression values. Then, HVGs were extracted using ModelVar function from scran, according to a significant deviation above the mean-variance fitted trend (BH-corrected *P*<0.05). These genes were selected for computing a PCA. The top 50 PCs were retained for batch correction using reducedMNN, which is an updated version of fastMNN. The resulting batch-corrected PCs were used for clustering and visualisation in downstream analysis.

### Predicting transcriptional identity and cell fate of primitive streak cells by mapping them against the atlas

Following the strategy to transfer annotations from the atlas to other datasets previously described above, cell type annotations were assigned to primitive streak cells according to the 15 nearest neighbours between the two manifolds (query data and atlas reference). The annotation transfer was performed separately for each batch to avoid introducing an extra confounding factor when applying MNN batch correction. Also, before mapping against the atlas, genes with total counts lower than 100 were filtered out from the Smart-seq2 datasets due to the large differences in dropouts. The resulting annotations were projected onto the UMAP extended atlas embryo landscape by highlighting the closest cells on it. By retaining the closest cell in the atlas and selecting the cell fate with highest probability allocated in a W-OT fates matrix, we aimed to not only map the observed transcriptome at E7.5 but make predictions about likely cell fates at future time points (e.g. E8.5 and E9.5).

### Primitive streak dissections and processing for Smart-seq

Mouse embryos were collected under the UK Home Office project licence number PP9552402. Animals used in this study were 6-16 week-old wild-type females of a mixed (CBAxC57BL/6) F2 background, maintained on a lighting regime of 14 h light and 10 h dark with food and water supplied *ad libitum*. Following timed mating crosses between transgenic mTmG [B6.129(Cg)-*Gt(ROSA)26Sor*^*tm4(ACTB*−*tdTomato*,*-EGFP)Luo*^/J (Muzumdar et al., 2007)] males and wild-type females, females were killed 7 days after a vaginal plug was observed via a Schedule 1 method. The uterine tissue and the decidua were cut away to extract the E7 embryo. The Reichert’s membrane was peeled off and embryos were staged according to Downs and Davies (1993). EB-stage embryos were taken for further analysis. For primitive streak dissections, the extra-embryonic part of the conceptus was cut off, the anterior and posterior embryonic portions were separated, and the posterior portion flattened out, taking care not to mix up the orientation. The posterior portion containing the primitive streak was cut at the halfway point and each half was halved again creating four similar-sized primitive streak segments. All embryo and primitive streak dissections were performed in Dulbecco’s PBS (with CaCl_2_ and MgCl_2_, Gibco) supplemented with 10% FCS (Gibco), 50 U/ml penicillin and 50 U/ml streptomycin.

For scRNA-seq of primitive streak cells, individual primitive streak regions were collected in separate Eppendorf tubes in FACS buffer [PBS without CaCl_2_ and MgCl_2_ (Gibco) supplemented with 10% FCS (Gibco), 50 U/ml penicillin and 50 U/ml streptomycin]. After centrifugation at 200 ***g*** for 5 min, tissues were resuspended in 200-250 μl of TrypLE Express (Life Technologies) and incubated at 37°C for 7 min with regular agitation and dissociated by pipetting. Cells were washed with 1 ml of FACS buffer, centrifuged at 200 ***g*** for 5 min and resuspended in FACS buffer with Hoechst 33358 for sorting. In order to reduce cell loss from these small cell populations, wild-type adult mouse thymocytes were added to the samples and were gated out based on their typical FSC-SSC profiles and absence of tdTomato expression. Each primitive streak region yielded on average 200 cells. Cells were kept on ice throughout the procedure. Individual cells were sorted directly into 96-well plates (Starlab, E1403-1200) into 2.3 µl of lysis buffer containing SUPERase-In RNase Inhibitor 20 U/µl (Ambion, AM2694), 10% Triton X-100 (Sigma-Aldrich, 93443) and RNAse-free water. Plates were processed using a combination of Smart-seq2 (Picelli et al., 2014) and mcSCRB-seq (Bagnoli et al., 2018). For detailed protocol see Sturgess et al. (2021). Pooled libraries were run on the Illumina HiSeq4000 at Cancer Research UK Cambridge Institute Genomics Core.

### Primitive streak grafts and static culture

mTmG transgenic embryos were dissected to harvest four equal primitive streak regions as described above. Each segment was deposited into a 50 μl dissection buffer drop on the inside lid of a 5 cm dish and labelled. One larger drop was deposited in the centre, into which up to five dissected wild-type embryos of the same stage (EB) were transferred. Primitive streak regions were grafted into orthotopic positions in the wild-type embryos using a pulled glass capillary needle attached to a mouth pipette. Cells from each donor region were divided over 2-3 recipient embryos with each recipient embryo receiving 50-100 primitive streak cells, mostly as a chain of cells rather than in suspension. Embryos were gently washed in a drop of culture medium and gently transferred into individual wells of a 96-well Ultra-Low adhesive plate (Corning) into 200 μl culture medium. Culture medium was 100% rat serum (Envigo, custom collected). Embryos were cultured at 37°C, 5% CO_2_ for 23-26 h.

### Fate contribution analysis

Embryos were taken out of the incubator and assessed for (the beginnings of) a heartbeat. Only embryos that lacked deformities and had a heartbeat were used for further analysis by microscopy or scRNA-seq to assess the fates of the grafted primitive streak cells. For microscopically observed fates, whole embryos/concepti were immunostained in U-bottom 96-well plates as follows. Whole embryos were fixed in 4% paraformaldehyde in PBS for 20-30 min at room temperature (RT) and washed three times in staining buffer (0.4% Triton X-100, 2% FCS in PBS). Embryos were incubated in individual Eppendorf tubes in staining buffer for 3-6 h at RT on a shaking rack. Reporter gene expression (mTom) was enhanced with anti-RFP antibody (Rockland, 600-401-279, 1:400) and embryonic vasculature visualised with anti-CD31 antibody (R&D Systems, AF3628, 1:200). Primary antibodies were diluted in the staining buffer and 50-100 μl used for each well/embryo. Incubation lasted overnight at 4°C, gently shaking. The next day, embryos were washed in staining buffer for up to 5-8 h at RT on a shaking rack. Whole mounts were then incubated with the secondary antibody solution (Alexa Fluor 555, Invitrogen, A31572, 1:200; Alexa Fluor 647, Invitrogen, A21447, 1:200) with nuclear dye (Hoechst 33358) overnight at 4°C and subsequently washed again for several hours or up to 24 h with several changes of the buffer. Wholemount embryos were imaged to assess overall contribution of mTom in relation to embryo anatomy (Zeiss 780 inverted confocal microscope at 10×). Embryos were next embedded in OCT and snap-frozen on ethanol-dry ice mix followed by cryosectioning, retaining every section to capture all mTom-expressing cells. Sections were then assessed, and notes taken of all areas with mTom^+^ contribution and careful analysis and scoring for detailed contribution to a selection of most anatomically distinct cell lineages (Table S8). Immunofluorescence in sections was imaged using a Zeiss 780 inverted confocal microscope with 25× (LD LCI PA 25×/0.8 DIC), 40× (LD C-Apochromat 40×/1.1) and 63× (Plan Apochromat 63×/1.4) objectives with oil immersion. Images were stored and analysed in OMERO. Adobe Photoshop was used for final image processing and Adobe Illustrator to generate figures.

## Supplementary Material

Supplementary Information

Table S1

Table S2

Table S3

Table S4

Table S5

Table S6

Table S7

Table S8

## Data Availability

Data from this study have been made available at https://marionilab.github.io/ExtendedMouseAtlas/, where links to both raw and processed data are found. Memory usage to process and analyse this data was optimised by means of *scarf*, a single-cell analysis framework (Dhapola et al., 2022).

## References

[R1] Aguilera-Castrejon A, Oldak B, Shani T, Ghanem N, Itzkovich C, Slomovich S, Tarazi S, Bayerl J, Chugaeva V, Ayyash M (2021). Ex utero mouse embryogenesis from pre-gastrulation to late organogenesis. Nature.

[R2] Argelaguet R, Clark SJ, Mohammed H, Stapel LC, Krueger C, Kapourani CA, Imaz-Rosshandler I, Lohoff T, Xiang Y, Hanna CW (2019). Multi-omics profiling of mouse gastrulation at single-cell resolution. Nature.

[R3] Bagnoli JW, Ziegenhain C, Janjic A, Wange LE, Vieth B, Parekh S, Geuder J, Hellmann I, Enard W (2018). Sensitive and powerful single-cell RNA sequencing using mcSCRB-seq. Nat Commun.

[R4] Baran Y, Bercovich A, Sebe-Pedros A, Lubling Y, Giladi A, Chomsky E, Meir Z, Hoichman M, Lifshitz A, Tanay A (2019). MetaCell: analysis of single-cell RNA-seq data using K-nn graph partitions. Genome Biol.

[R5] Barile M, Imaz-Rosshandler I, Inzani I, Ghazanfar S, Nichols J, Marioni JC, Guibentif C, Göttgens B (2021). Coordinated changes in gene expression kinetics underlie both mouse and human erythroid maturation. Genome Biol.

[R6] Bowling S, Sritharan D, Osorio FG, Nguyen M, Cheung P, Rodriguez-Fraticelli A, Patel S, Yuan WC, Fujiwara Y, Li BE (2020). An engineered CRISPR-Cas9 mouse line for simultaneous readout of lineage histories and gene expression profiles in single cells. Cell.

[R7] Cao J, Spielmann M, Qiu X, Huang X, Ibrahim DM, Hill AJ, Zhang F, Mundlos S, Christiansen L, Steemers FJ (2019). The single-cell transcriptional landscape of mammalian organogenesis. Nature.

[R8] Chan MM, Smith ZD, Grosswendt S, Kretzmer H, Norman TM, Adamson B, Jost M, Quinn JJ, Yang D, Jones MG (2019). Molecular recording of mammalian embryogenesis. Nature.

[R9] Clark SJ, Argelaguet R, Lohoff T, Krueger F, Drage D, Göttgens B, Marioni JC, Nichols J, Reik W (2022). Single-cell multi-omics profiling links dynamic DNA methylation to cell fate decisions during mouse early organogenesis. Genome Biol.

[R10] Conrad Hal Waddington HK (1957). The Strategy of the Genes: A Discussion of Some Aspects of Theoretical Biology.

[R11] Davidson D (1989). Genet Res.

[R12] de Bruijn M, Dzierzak E (2017). Runx transcription factors in the development and function of the definitive hematopoietic system. Blood.

[R13] de Soysa TY, Ranade SS, Okawa S, Ravichandran S, Huang Y, Salunga HT, Schricker A, Del Sol A, Gifford CA, Srivastava D (2019). Single-cell analysis of cardiogenesis reveals basis for organ-level developmental defects. Nature.

[R14] Dhapola P, Rodhe J, Olofzon R, Bonald T, Erlandsson E, Soneji S, Karlsson G (2022). Scarf enables a highly memory-efficient analysis of large-scale single-cell genomics data. Nat Commun.

[R15] Dobin A, Davis CA, Schlesinger F, Drenkow J, Zaleski C, Jha S, Batut P, Chaisson M, Gingeras TR (2013). STAR: ultrafast universal RNA-seq aligner. Bioinformatics.

[R16] Downs KM, Davies T (1993). Staging of gastrulating mouse embryos by morphological landmarks in the dissecting microscope. Development.

[R17] Elsaid R, Soares-Da-Silva F, Peixoto M, Amiri D, Mackowski N, Pereira P, Bandeira A, Cumano A (2020). Hematopoiesis: a layered organization across chordate species. Front Cell Dev Biol.

[R18] Ghazanfar S, Guibentif C, Marioni JC (2023). Stabilized mosaic single-cell data integration using unshared features. Nat Biotechnol.

[R19] Gittins R (1985). Canonical Analysis; a Review with Applications in Ecology.

[R20] Grosswendt S, Kretzmer H, Smith ZD, Kumar AS, Hetzel S, Wittler L, Klages S, Timmermann B, Mukherji S, Meissner A (2020). Epigenetic regulator function through mouse gastrulation. Nature.

[R21] Gu Z, Eils R, Schlesner M (2016). Complex heatmaps reveal patterns and correlations in multidimensional genomic data. Bioinformatics.

[R22] Guibentif C, Griffiths JA, Imaz-Rosshandler I, Ghazanfar S, Nichols J, Wilson V, Göttgens B, Marioni JC (2021). Diverse routes toward early somites in the mouse embryo. Dev Cell.

[R23] Han X, Wang R, Zhou Y, Fei L, Sun H, Lai S, Saadatpour A, Zhou Z, Chen H, Ye F (2018). Mapping the mouse cell atlas by Microwell-Seq. Cell.

[R24] Hao Y, Hao S, Andersen-Nissen E, Mauck WM, Zheng S, Butler A, Lee MJ, Wilk AJ, Darby C, Zager M (2021). Integrated analysis of multimodal single-cell data. Cell.

[R25] He P, Williams BA, Trout D, Marinov GK, Amrhein H, Berghella L, Goh ST, Plajzer-Frick I, Afzal V, Pennacchio LA (2020). The changing mouse embryo transcriptome at whole tissue and single-cell resolution. Nature.

[R26] Ibarra-Soria X, Jawaid W, Pijuan-Sala B, Ladopoulos V, Scialdone A, Jörg DJ, Tyser RCV, Calero-Nieto FJ, Mulas C, Nichols J (2018). Defining murine organogenesis at single-cell resolution reveals a role for the leukotriene pathway in regulating blood progenitor formation. Nat Cell Biol.

[R27] Jacomy M, Venturini T, Heymann S, Bastian M (2014). ForceAtlas2, a continuous graph layout algorithm for handy network visualization designed for the Gephi software. PLoS One.

[R28] Jin S, Guerrero-Juarez CF, Zhang L, Chang I, Ramos R, Kuan CH, Myung P, Plikus MV, Nie Q (2021). Inference and analysis of cell-cell communication using CellChat. Nat Commun.

[R29] Kanti Mardia JK, Bibby J (1979). Multivariate Analysis.

[R30] Kinder SJ, Tsang TE, Quinlan GA, Hadjantonakis AK, Nagy A, Tam PP (1999). The orderly allocation of mesodermal cells to the extraembryonic structures and the anteroposterior axis during gastrulation of the mouse embryo. Development.

[R31] La Manno G, Siletti K, Furlan A, Gyllborg D, Vinsland E, Mossi Albiach A, Mattsson Langseth C, Khven I, Lederer AR, Dratva LM (2021). Molecular architecture of the developing mouse brain. Nature.

[R32] Lescroart F, Wang X, Lin X, Swedlund B, Gargouri S, Sànchez-Dànes A, Moignard V, Dubois C, Paulissen C, Kinston S (2018). Defining the earliest step of cardiovascular lineage segregation by single-cell RNA-seq. Science.

[R33] Lun AT, Mccarthy DJ, Marioni JC (2016). A step-by-step workflow for low-level analysis of single-cell RNA-seq data with Bioconductor. F1000Res.

[R34] Lupu I-E, Kirschnick N, Weischer S, Martinez-Corral I, Forrow A, Lahmann I, Riley PR, Zobel T, Makinen T, Kiefer F (2022). Direct specification of lymphatic endothelium from non-venous angioblasts. bioRxiv.

[R35] McGrath KE, Frame JM, Fegan KH, Bowen JR, Conway SJ, Catherman SC, Kingsley PD, Koniski AD, Palis J (2015). Distinct sources of hematopoietic progenitors emerge before HSCs and provide functional blood cells in the mammalian embryo. Cell Rep.

[R36] Mittnenzweig M, Mayshar Y, Cheng S, Ben-Yair R, Hadas R, Rais Y, Chomsky E, Reines N, Uzonyi A, Lumerman L (2021). A single-embryo, single-cell time-resolved model for mouse gastrulation. Cell.

[R37] Mohammed H, Hernando-Herraez I, Savino A, Scialdone A, Macaulay I, Mulas C, Chandra T, Voet T, Dean W, Nichols J (2017). Single-cell landscape of transcriptional heterogeneity and cell fate decisions during mouse early gastrulation. Cell Rep.

[R38] Monge G (1781). Mémoire sur la théorie des déblais et des remblais. Histoire del’Académie Royale des Sciences de Paris, avec les Mémoires de Mathématique et de Physique pour la même anné.

[R39] Murayama E, Vivier C, Schmidt A, Herbomel P (2023). Alcam-a and Pdgfr-α are essential for the development of sclerotome-derived stromal cells that support hematopoiesis. Nat Commun.

[R40] Muzumdar MD, Tasic B, Miyamichi K, Li L, Luo L (2007). A global double-fluorescent Cre reporter mouse. Genesis.

[R41] Nguyen PD, Hollway GE, Sonntag C, Miles LB, Hall TE, Berger S, Fernandez KJ, Gurevich DB, Cole NJ, Alaei S (2014). Haematopoietic stem cell induction by somite-derived endothelial cells controlled by meox1. Nature.

[R42] Nowotschin S, Setty M, Kuo YY, Liu V, Garg V, Sharma R, Simon CS, Saiz N, Gardner R, Boutet SC (2019). The emergent landscape of the mouse gut endoderm at single-cell resolution. Nature.

[R43] Pardanaud L, Luton D, Prigent M, Bourcheix LM, Catala M, Dieterlen-Lievre F (1996). Two distinct endothelial lineages in ontogeny, one of them related to hemopoiesis. Development.

[R44] Persad S, Choo ZN, Dien C, Sohail N, Masilionis I, Chaligné R, Nawy T, Brown CC, Sharma R, Pe’er I (2023). SEACells infers transcriptional and epigenomic cellular states from single-cell genomics data. Nat Biotechnol.

[R45] Picelli S, Faridani OR, Björklund AK, Winberg G, Sagasser S, Sandberg R (2014). Full-length RNA-seq from single cells using Smart-seq2. Nat Protoc.

[R46] Pijuan-Sala B, Griffiths JA, Guibentif C, Hiscock TW, Jawaid W, Calero-Nieto FJ, Mulas C, Ibarra-Soria X, Tyser RCV, Ho DLL (2019). A single-cell molecular map of mouse gastrulation and early organogenesis. Nature.

[R47] Polanski K, Young MD, Miao Z, Meyer KB, Teichmann SA, Park JE (2020). BBKNN: fast batch alignment of single cell transcriptomes. Bioinformatics.

[R48] Pouget C, Gautier R, Teillet MA, Jaffredo T (2006). Somite-derived cells replace ventral aortic hemangioblasts and provide aortic smooth muscle cells of the trunk. Development.

[R49] Qiu C, Cao J, Martin BK, Li T, Welsh IC, Srivatsan S, Huang X, Calderon D, Noble WS, Disteche CM (2022). Systematic reconstruction of cellular trajectories across mouse embryogenesis. Nat Genet.

[R50] Qiu C, Martin BK, Welsh IC, Daza RM, Le T-M, Huang X, Nichols EK, Taylor ML, Fulton O, O’day DR (2023). A single-cell transcriptional timelapse of mouse embryonic development, from gastrula to pup. bioRxiv.

[R51] Ritchie ME, Phipson B, Wu D, Hu Y, Law CW, Shi W, Smyth GK (2015). limma powers differential expression analyses for RNA-sequencing and microarray studies. Nucleic Acids Res.

[R52] Rodriguez AM, Jin DX, Wolfe AD, Mikedis MM, Wierenga L, Hashmi MP, Viebahn C, Downs KM (2017). Brachyury drives formation of a distinct vascular branchpoint critical for fetal-placental arterial union in the mouse gastrula. Dev Biol.

[R53] Rybtsov S, Batsivari A, Bilotkach K, Paruzina D, Senserrich J, Nerushev O, Medvinsky A (2014). Tracing the origin of the HSC hierarchy reveals an SCF-dependent, IL-3-independent CD43(-) embryonic precursor. Stem Cell Rep.

[R54] Saelens W, Cannoodt R, Todorov H, Saeys Y (2019). A comparison of single-cell trajectory inference methods. Nat Biotechnol.

[R55] Sato Y, Watanabe T, Saito D, Takahashi T, Yoshida S, Kohyama J, Ohata E, Okano H, Takahashi Y (2008). Notch mediates the segmental specification of angioblasts in somites and their directed migration toward the dorsal aorta in avian embryos. Dev Cell.

[R56] Schiebinger G, Shu J, Tabaka M, Cleary B, Subramanian V, Solomon A, Gould J, Liu S, Lin S, Berube P (2019). Optimal-transport analysis of single-cell gene expression identifies developmental trajectories in reprogramming. Cell.

[R57] Scialdone A, Tanaka Y, Jawaid W, Moignard V, Wilson NK, Macaulay IC, Marioni JC, Göttgens B (2016). Resolving early mesoderm diversification through single-cell expression profiling. Nature.

[R58] Street K, Risso D, Fletcher RB, Das D, Ngai J, Yosef N, Purdom E, Dudoit S (2018). Slingshot: cell lineage and pseudotime inference for single-cell transcriptomics. BMC Genomics.

[R59] Sturgess KHM, Calero-Nieto FJ, Göttgens B, Wilson NK (2021). Single-cell analysis of hematopoietic stem cells. Methods Mol Biol.

[R60] Subelj L, Bajec M (2011). Unfolding communities in large complex networks: combining defensive and offensive label propagation for core extraction. Phys Rev E Stat Nonlin Soft Matter Phys.

[R61] Sun J, Ting MC, Ishii M, Maxson R (2016). Msx1 and Msx2 function together in the regulation of primordial germ cell migration in the mouse. Dev Biol.

[R62] Tam PPL, Ho JWK (2020). Cellular diversity and lineage trajectory: insights from mouse single cell transcriptomes. Development.

[R63] Tani S, Chung UI, Ohba S, Hojo H (2020). Understanding paraxial mesoderm development and sclerotome specification for skeletal repair. Exp Mol Med.

[R64] Tober J, Koniski A, Mcgrath KE, Vemishetti R, Emerson R, De Mesy-Bentley KK, Waugh R, Palis J (2007). The megakaryocyte lineage originates from hemangioblast precursors and is an integral component both of primitive and of definitive hematopoiesis. Blood.

[R65] Traag VA, Waltman L, Van Eck NJ (2019). From Louvain to Leiden: guaranteeing well-connected communities. Sci Rep.

[R66] Tritschler S, Büttner M, Fischer DS, Lange M, Bergen V, Lickert H, Theis FJ (2019). Concepts and limitations for learning developmental trajectories from single cell genomics. Development.

[R67] Van den Berge K, Roux De Bezieux H, Street K, Saelens W, Cannoodt R, Saeys Y, Dudoit S, Clement L (2020). Trajectory-based differential expression analysis for single-cell sequencing data. Nat Commun.

[R68] Yamanaka Y, Hamidi S, Yoshioka-Kobayashi K, Munira S, Sunadome K, Zhang Y, Kurokawa Y, Ericsson R, Mieda A, Thompson JL (2023). Reconstituting human somitogenesis in vitro. Nature.

[R69] Yokomizo T, Watanabe N, Umemoto T, Matsuo J, Harai R, Kihara Y, Nakamura E, Tada N, Sato T, Takaku T (2019). Hlf marks the developmental pathway for hematopoietic stem cells but not for erythro-myeloid progenitors. J Exp Med.

[R70] Yvernogeau L, Gautier R, Petit L, Khoury H, Relaix F, Ribes V, Sang H, Charbord P, Souyri M, Robin C (2019). In vivo generation of haematopoietic stem/progenitor cells from bone marrow-derived haemogenic endothelium. Nat Cell Biol.

[R71] Yvernogeau L, Klaus A, Rooijen CV, Robin C (2020). Generation of a new Tbx6-inducible reporter mouse line to trace presomitic mesoderm derivatives throughout development and in adults. bioRxiv.

[R72] Zhou Y, Zhou B, Pache L, Chang M, Khodabakhshi AH, Tanaseichuk O, Benner C, Chanda SK (2019). Metascape provides a biologist-oriented resource for the analysis of systems-level datasets. Nat Commun.

