# Supplementary material for: Tracking early mammalian organogenesis – prediction and validation of differentiation trajectories at whole organism scale
Source: Development. Author manuscript; Available in PMC 2024 Mar 6. (PMC10906099; doi:10.1242/dev.201867)
Supplement: Supplementary Information [file EMS194370-supplement-Supplementary_Information.pdf]

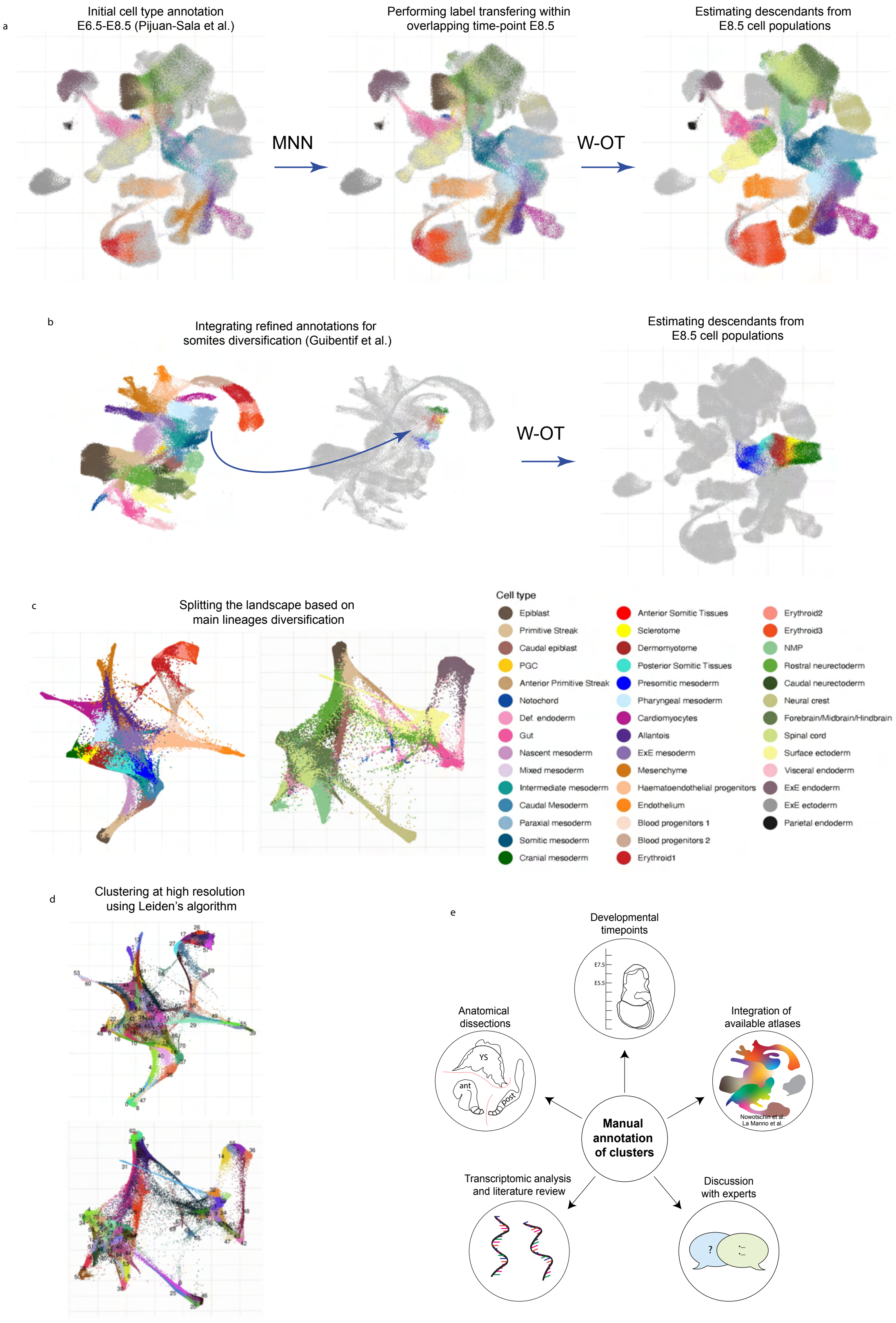

Development • Supplementary information

Fig. S1. Extending cell type annotations step by step.

a) UMAP layouts are used to visualise the first stage of cell type annotation, cells are coloured by cell type [Pijuan-Sala et al. 2019] but light grey cells represent those new that have not yet been annotated. Initially, mutual nearest neighbours (MNN) were used to transfer E8.5 cell type annotations from [Pijuan-Sala et al. 2019] to newly profiled cells within the overlapping time-point. Next, cell descendants are estimated for all existing E8.5 cell populations using W-OT by pushing forward the mass over the transport maps in the following time-points (E8.75-E9.5). Here, only cell descendant populations are coloured by cell type and light grey cells refer instead, to those from the original atlas (E6.5-E8.5). **b)** Refined cell type annotations for Paraxial and Somitic mesoderm from Guibentif et al. [Pijuan-Sala et al. 2019] are integrated through the same process described in a). **c)** The atlas is splitted into two landscapes based on the annotations deriving from a) and b). The mesodermal landscape at the left and the ectodermal/ endodermal one at the right. Cells are coloured by original Atlas cell types and descendants. **d)** Cells are coloured by high resolution louvain clustering as the starting point of new cell type annotations. **e)** Highly variable genes, batch correction and leiden clustering was performed independently for the two landscapes obtained from C. These clusters are manually annotated using different sources of information as illustrated in the right side scheme. For instance, cell type annotations from other atlases such as [Nowotschin et al. 2019 and La Manno et. al, 2021] were used as guidance, differential expression analysis and literature marker inspection and trajectory analysis.

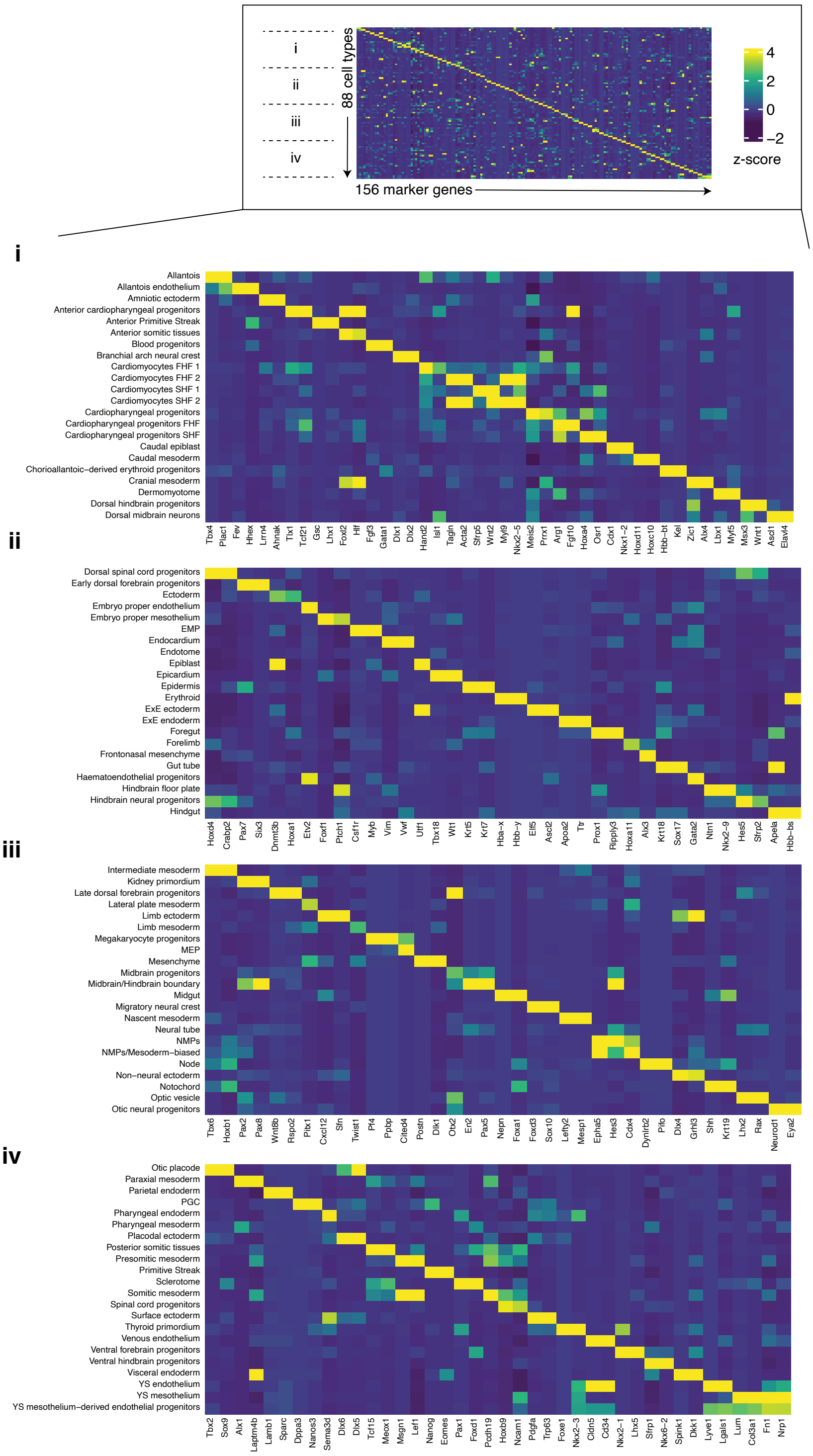

Fig. S2. Cell type marker genes.
Collection of gene markers (~ 2 genes/cell type) for the 88 cell types shown as a complement for Figure 1. Mean gene expression values were computed and scaled by rows (Z-score), cell types are listed alphabetically.

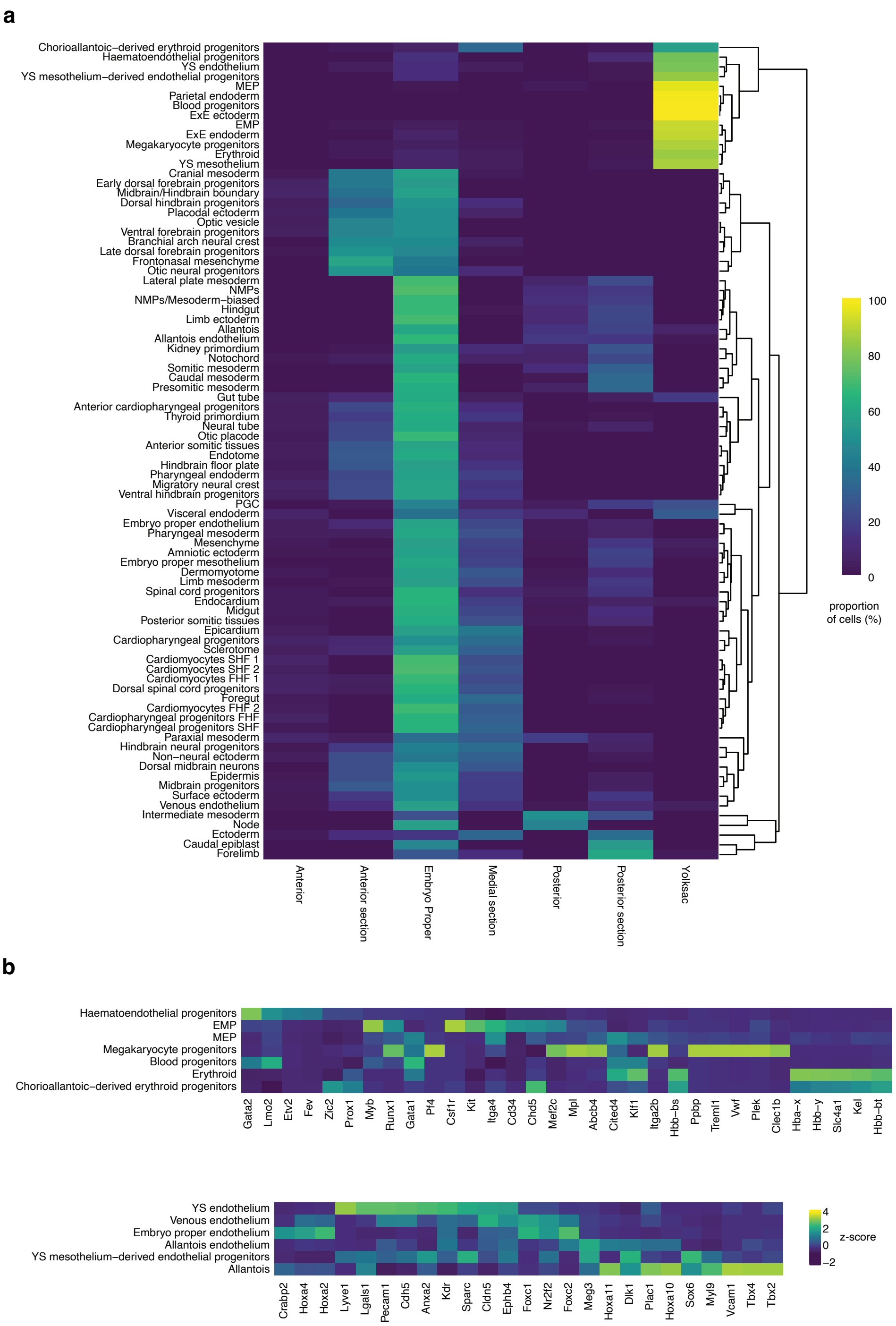

Fig. S3. Cell type distribution in different anatomical regions.

(a) Heatmap showing the proportion of cells (%) in different anatomical regions, normalized per row (cell type). Rows are hierarchically clustered and only cells from E8.5-E9.5 sub-dissected embryos are included. **(b)** Collection of gene markers for endothelium, haemato-endothelial and blood progenitors shown as a complement for Figure 2. Mean gene expression values were computed and scaled by rows (Z-score).

Development • Supplementary information

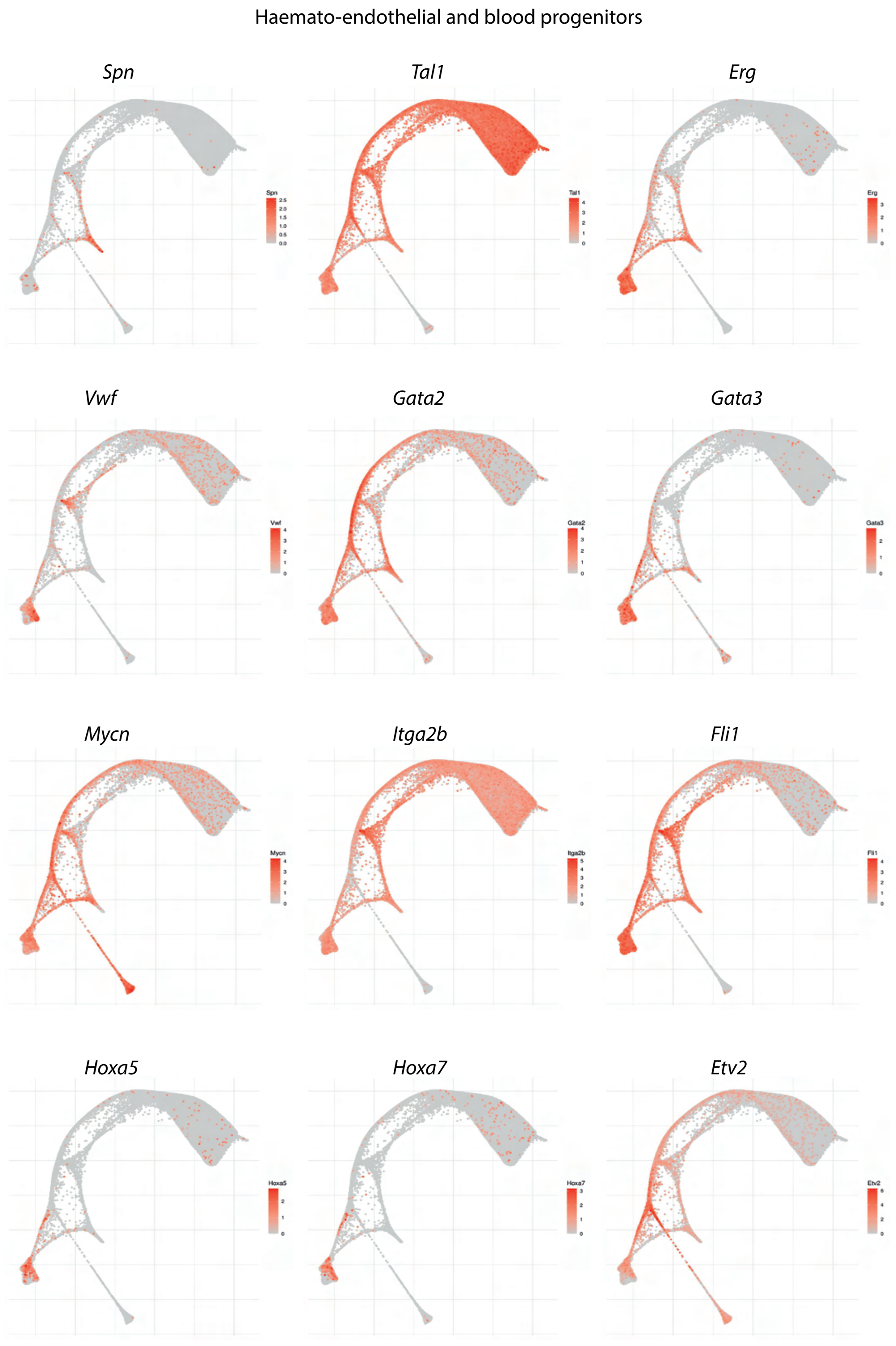

Fig. S4. Haemato-endothelial and blood progenitors.
Collection of gene markers for haemato-endothelial and blood progenitors shown as a complement for Figure 2. Force directed layouts displaying gene markers expression levels.

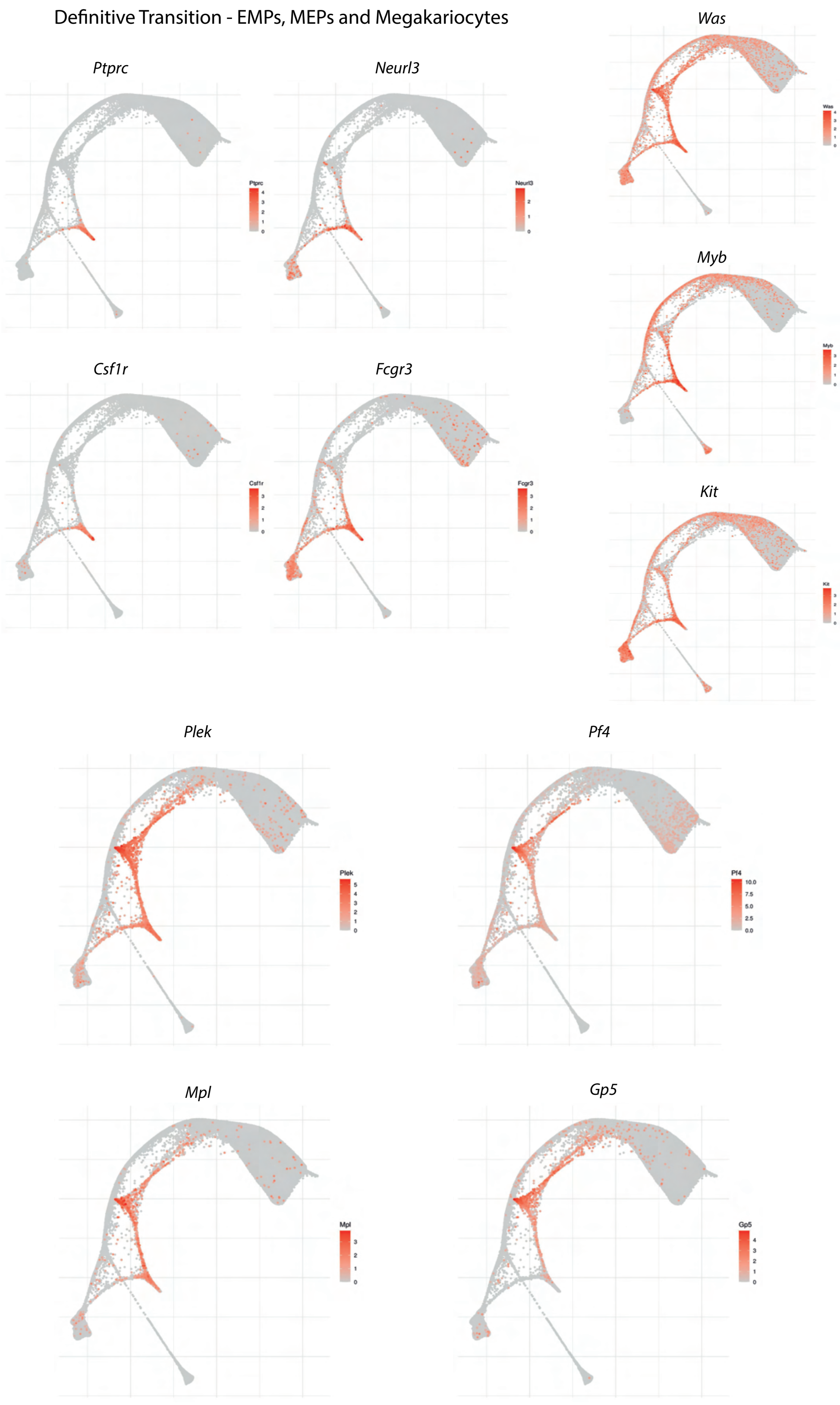

Fig. S5. Definitive transition - EMPs, MEPs and megakaryocytes.
Collection of gene markers for definitive blood populations shown as a complement for Figure 2. Force directed layouts displaying gene markers expression levels.

Definitive Transition - Myeloid progenitors

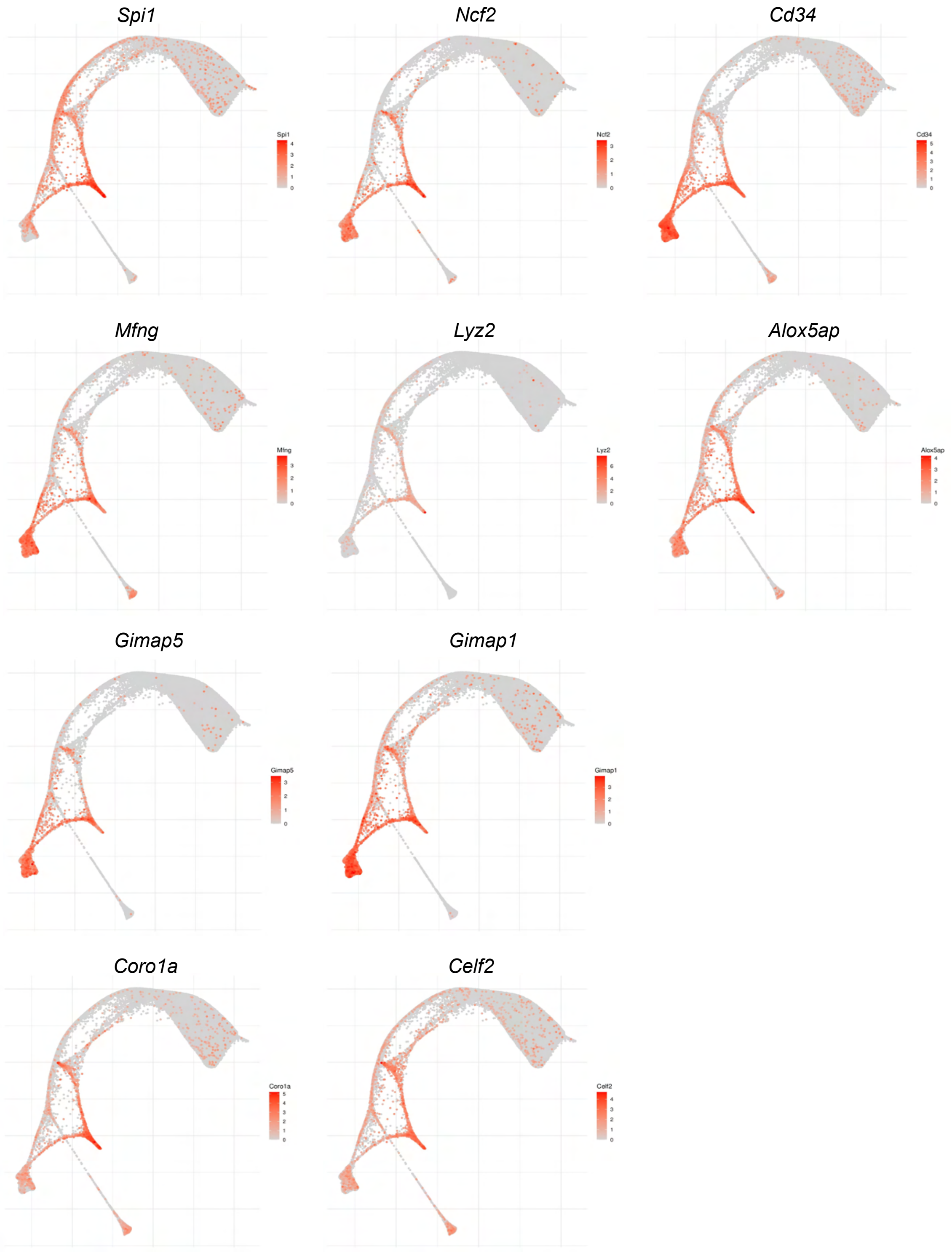

Fig. S6. Definitive transition - Myeloid progenitors.
Collection of gene markers for definitive blood populations shown as a complement for Figure 2. Force directed layouts displaying gene markers expression levels.

Development • Supplementary information

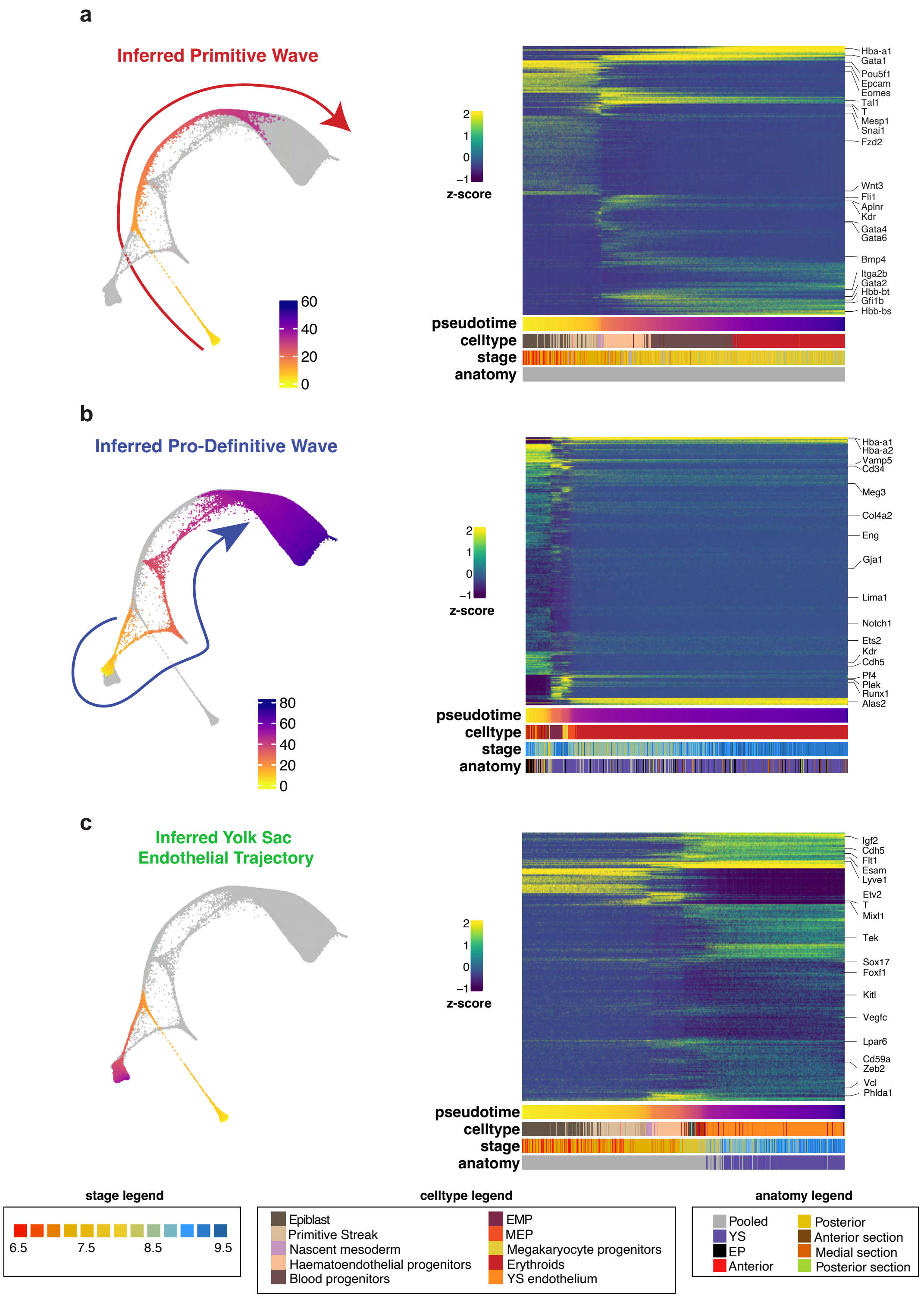

Development • Supplementary information

Fig. S7. Gene expression changes along the inferred primitive and definitive YS waves of blood and YS endothelial formation.

Force directed layout showing the subset of cells contributing to the primitive **(a)** definitive YS **(b)** and YS endothelial inferred differentiation trajectories **(c)** coloured by pseudotime. Heat maps display the top 300 genes associated with the pseudotimes for the various inferred trajectories (Tradeseq associationTest, $p<0.01$ and meanLogFC > 2). Rows (genes) are hierarchically clustered together. Accompanying metadata including cell type annotation, stage and anatomy is displayed below the heatmap of gene expression. Mean gene expression values were computed and scaled by rows (Z-score).

Development • Supplementary information

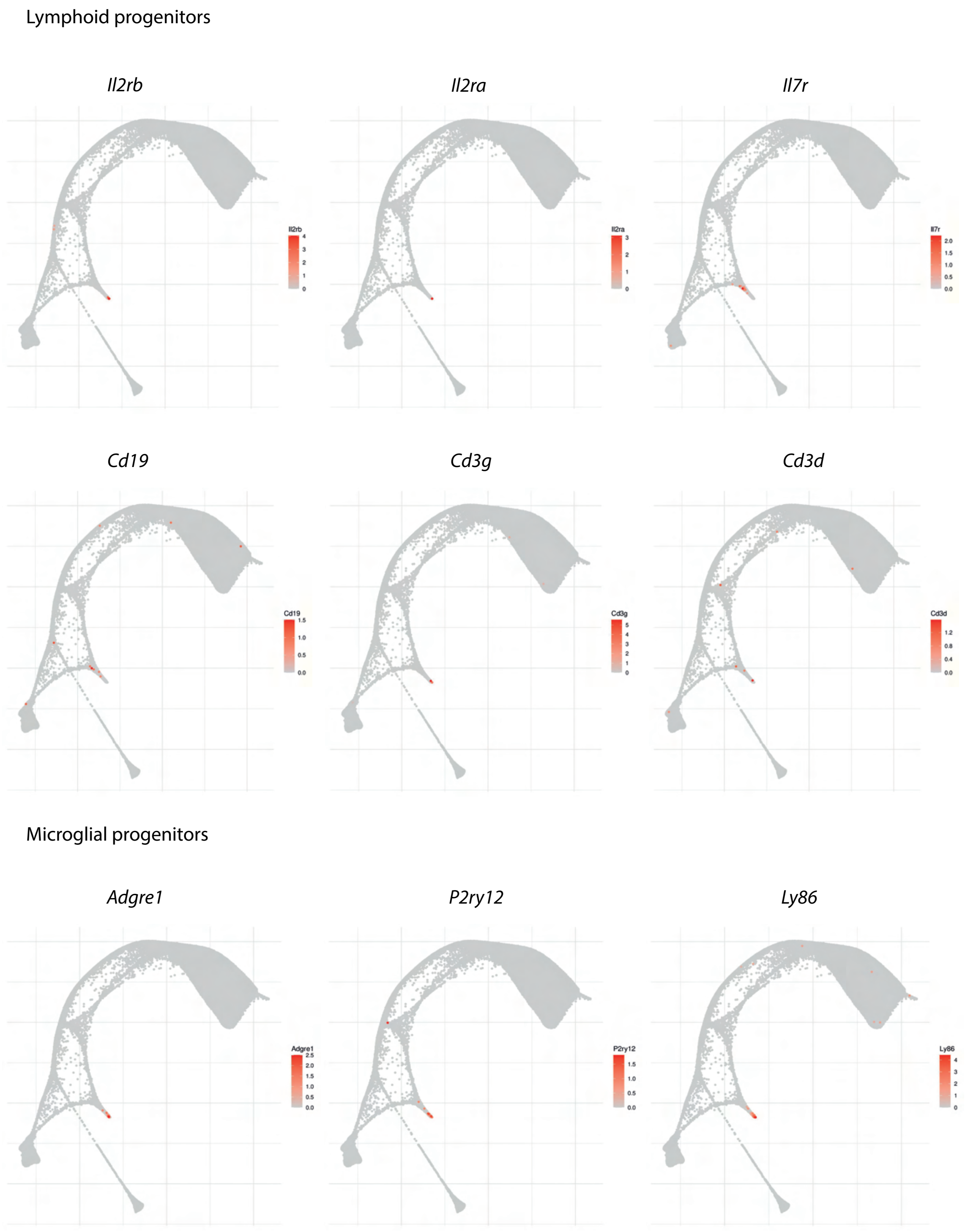

Fig. S8. Definitive transition - Lymphoid and microglial progenitors.
Collection of gene markers for definitive blood populations shown as a complement for Figure 2. Force directed layouts displaying gene markers expression levels.

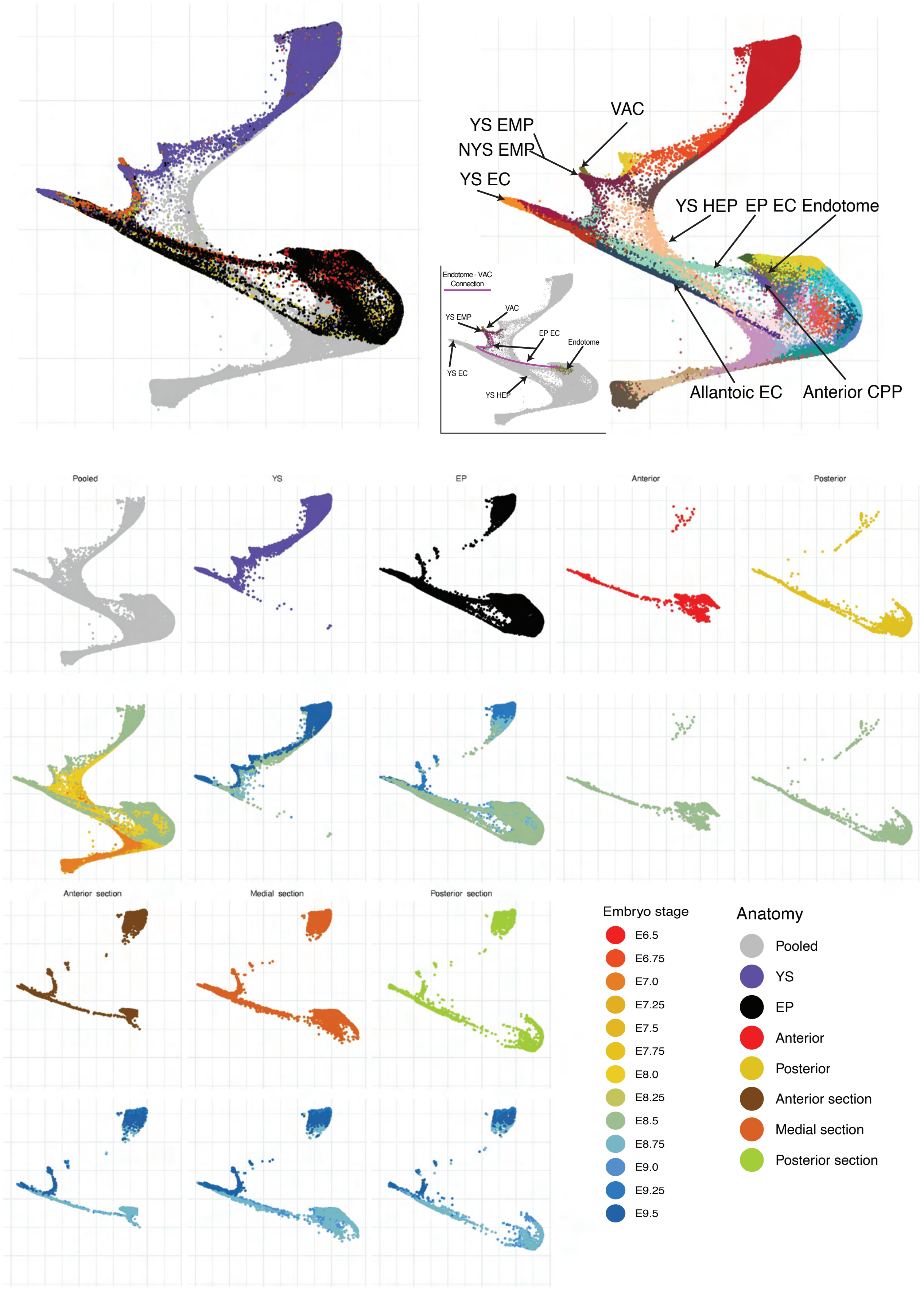

VAC
YS EMP
NYS EMP
YS EC
YS HEP
EP EC
Endotome
Allantoic EC
Anterior CPP
Endotome - VAC Connection
Pooled
YS
EP
Anterior
Posterior
Anterior section
Medial section
Posterior section
Embryo stage
E6.5
E6.75
E7.0
E7.25
E7.5
E7.75
E8.0
E8.25
E8.5
E8.75
E9.0
E9.25
E9.5
Anatomy
Pooled
YS
EP
Anterior
Posterior
Anterior section
Medial section
Posterior section

Fig. S9. Diverse anatomical origins of the haemato-endothelial landscape. Force directed layout of the haemato-endothelial landscape highlighting anatomical locations and relevant cell type populations (top); each anatomical region is shown in a different panel (bottom). Here, cells are coloured by anatomical locations as well as by embryo stage. Note that embryo proper sub dissections where no distinction between anterior, medial and posterior was made before sequencing are labelled as EP.

Development • Supplementary information

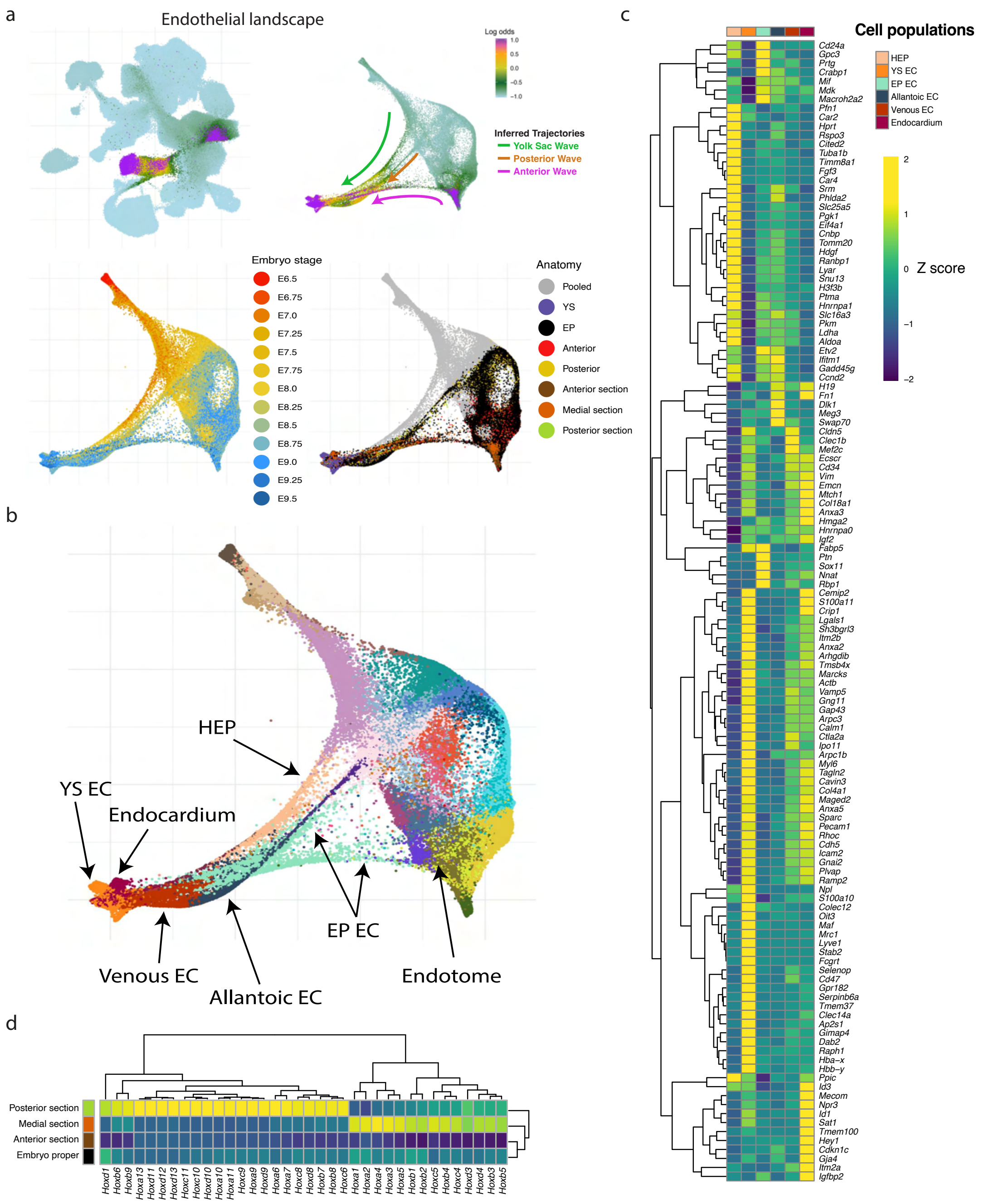

Development • Supplementary information

Fig. S10. Different origins of endothelial cells.

UMAP layout of the mouse extended atlas displaying the log odds of fate probabilities exclusively associated with endothelial populations (top left). Cells with log odds > - 1 were retained to generate a force directed layout. Cells are coloured by Log odds of fate probabilities of endothelial cells, embryo stage and anatomical region. Arrows highlight three putative endothelial differentiation trajectories. **b)** Force directed layout of the endothelial landscape highlighting relevant cell populations. HEP: Haemato-endothelial progenitors. EC: Endothelial cell. **c)** Heat map of differentially expressed genes across distinct endothelial populations. Mean gene expression values were computed and scaled by rows (Z-score). **(d)** Heat map showing Hox gene expression of embryo proper endothelial cells from distinct anatomical locations. Mean gene expression values were computed and scaled by columns (Z-score).

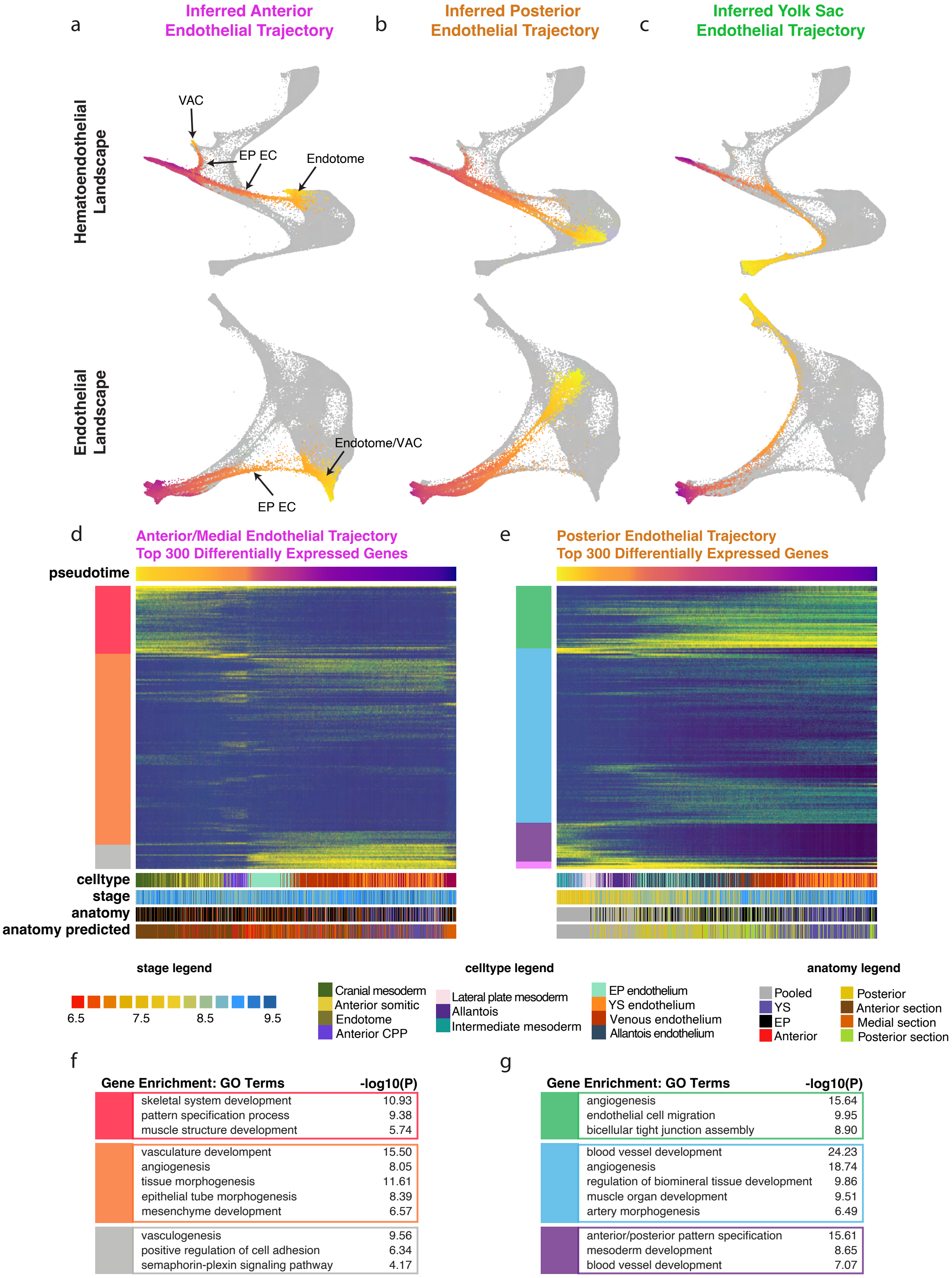

Development • Supplementary information

Fig. S11. Gene expression changes along the inferred anterior and posterior endothelial differentiation trajectories.
(a) Force directed layouts of the hematoendothelial (top row) and endothelial landscapes (bottow row) showing the predicted subset of cells contributing to the anterior/medial (left column), posterior (middle column) and YS endothelial inferred differentiation trajectories coloured by pseudotime. **(b,c)** Heat maps displaying the top 300 genes associated with the anterior/medial **(d)** and posterior **(e)** inferred endothelial differentiation trajectories (Tradeseq associationTest, $p<0.01$ and meanLogFC > 2). Rows (genes) are hierarchically clustered together. Accompanying metadata including cell type annotations, stage, anatomy and predicted anatomy is displayed below the heatmap of gene expression. Mean gene expression values were computed and scaled by rows (Z-score). **(f,g)** Gene Ontology Biological Process term enrichment analysis was performed using Metascape on clusters of genes **(f: red, orange, grey; g: green, blue, purple, pink)** highlighted by the different colour blocks shown on the left of the heatmaps.

Development • Supplementary information

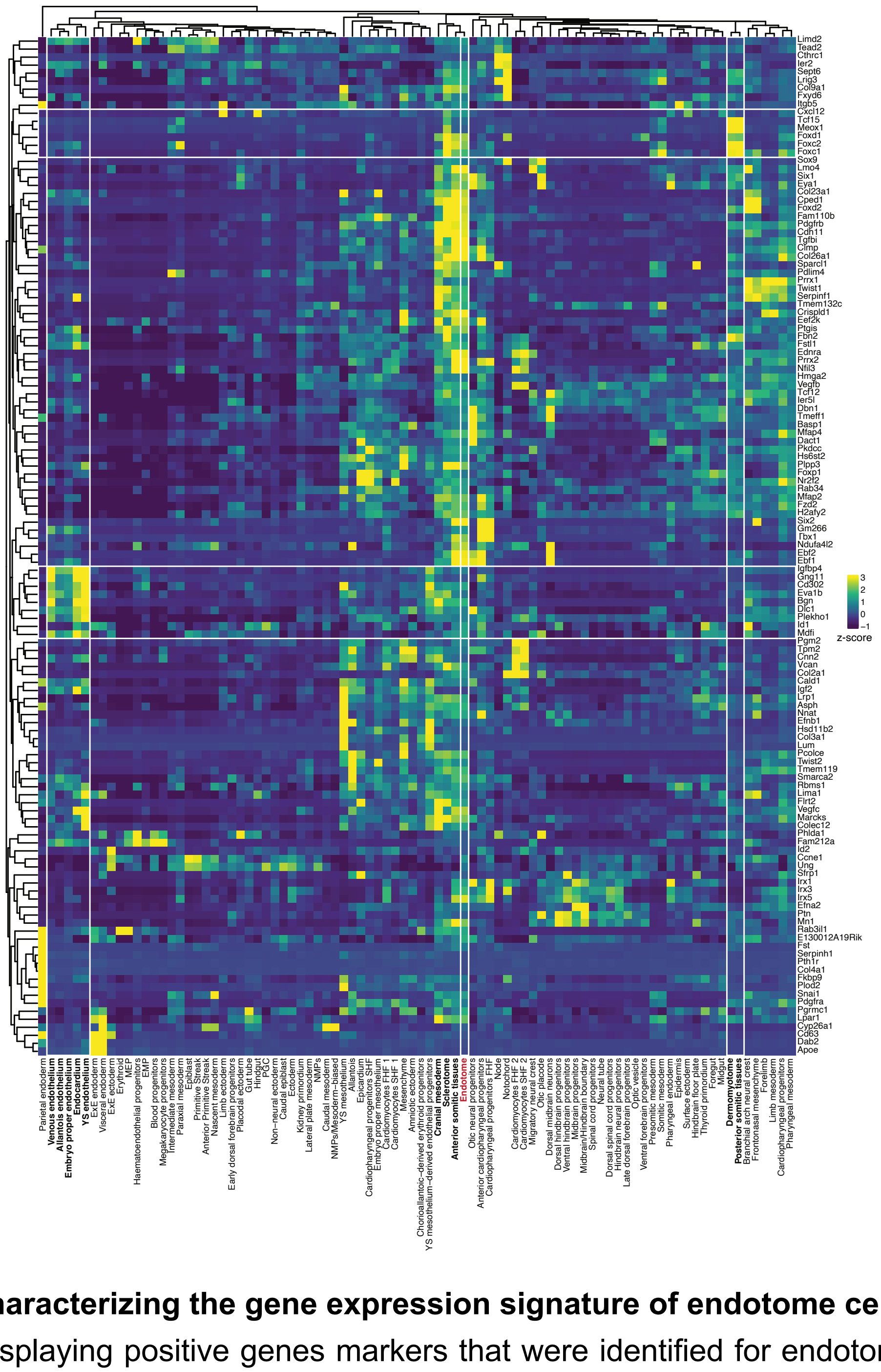

Fig. S12. Characterizing the gene expression signature of endotome cells. Heat map displaying positive genes markers that were identified for endotome cells (test = wilcox, logfc_threshold = 0.25, min_pct = 0.25), cell types (columns) and marker genes (rows) are clustered hierarchically. Mean gene expression values were computed and scaled by rows (Z-score). White boxes highlight endotome marker genes that are also expressed by endothelial cells or dermomyotome cell populations.

Development • Supplementary information

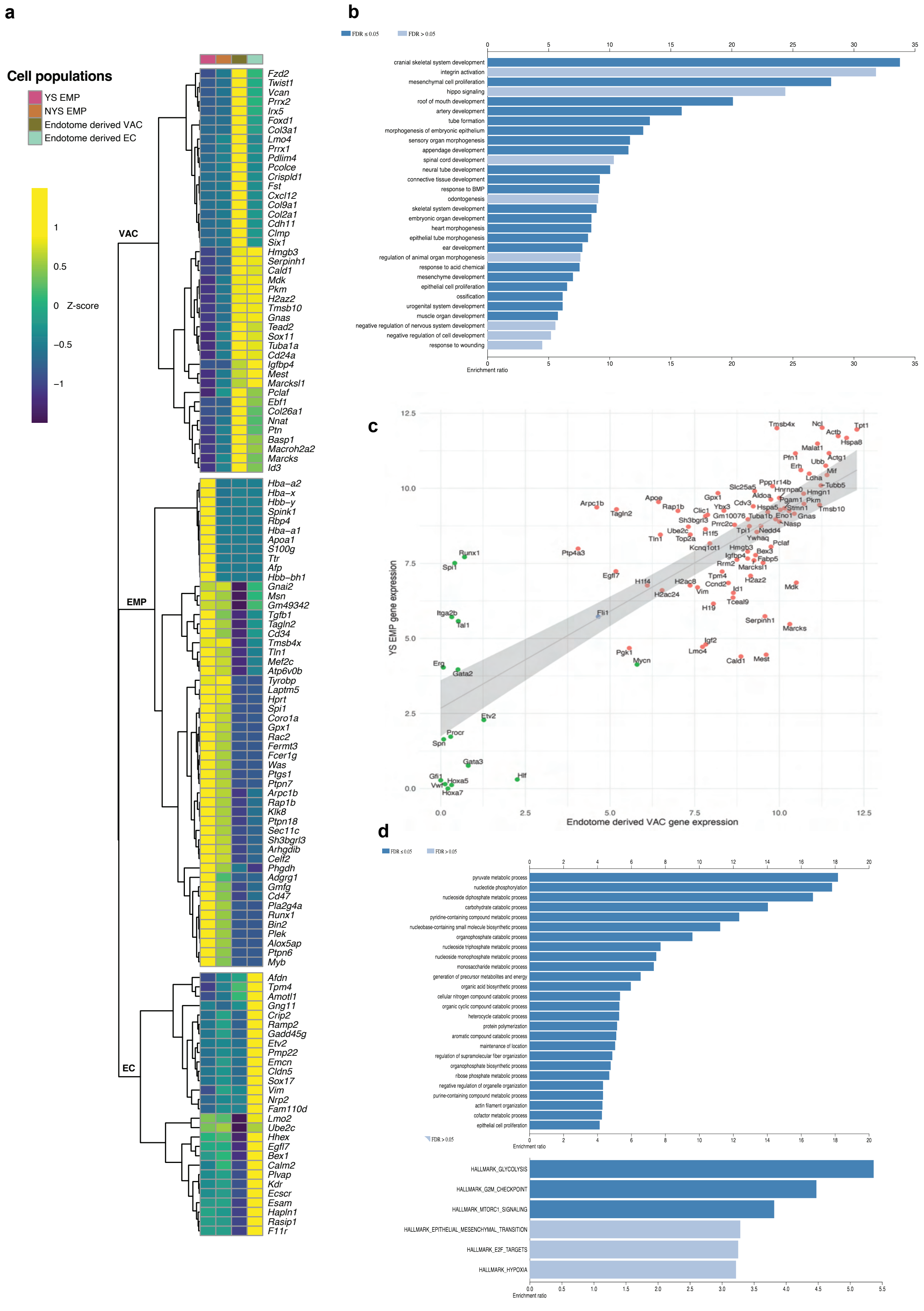

Development • Supplementary information

Fig. S13. Gene expression signature of endotome and VAC populations.

a) Heat map displaying differentially expressed genes across different cell populations clustered together in the region highlighted in Fig.3. Dii. YS EMP: Yolk Sack EMP, NYS EMP: Non-Yolk Sack EMP, Endotome derived VAC: Endotome derived vascular associated cells (This heat map is an extended version of the analysis performed for Fig.3.E). Mean gene expression values were computed and scaled by rows (Z-score). **b)** Gene set over representation analysis (ORA) performed with *webgestalt*[Liao et al., 2019] against Gene Ontology Biological Processes of VAC genes highlighted in panel A. Significant terms (FDR < 0.1) are generally associated with connective tissue development. **c)** Gene correlation analysis between YS EMPs and endotome derived VACs (red dots: positively correlated genes identified by CCA, green dots: manually selected genes associated with HSC progenitors, blue dots: Intersecting genes between both). Metacells were used to strengthen the gene expression signals (see methods). **d)** ORA of positively correlated genes against hallmark gene sets (FDR < 0.1) and Gene Ontology Biological Processes (FDR < 0.05). The resulting hallmarks reflect cell growth (evidenced by cell cycle genes, mTORC1 signalling and E2f2 targets), glycolytic metabolism and *Myc* activation (the latter above the significance threshold) as well as epithelial-mesenchymal transition while no evidence of haematopoietic identity, but leaving the possibility of niche function akin to the situation observed in Zebrafish.

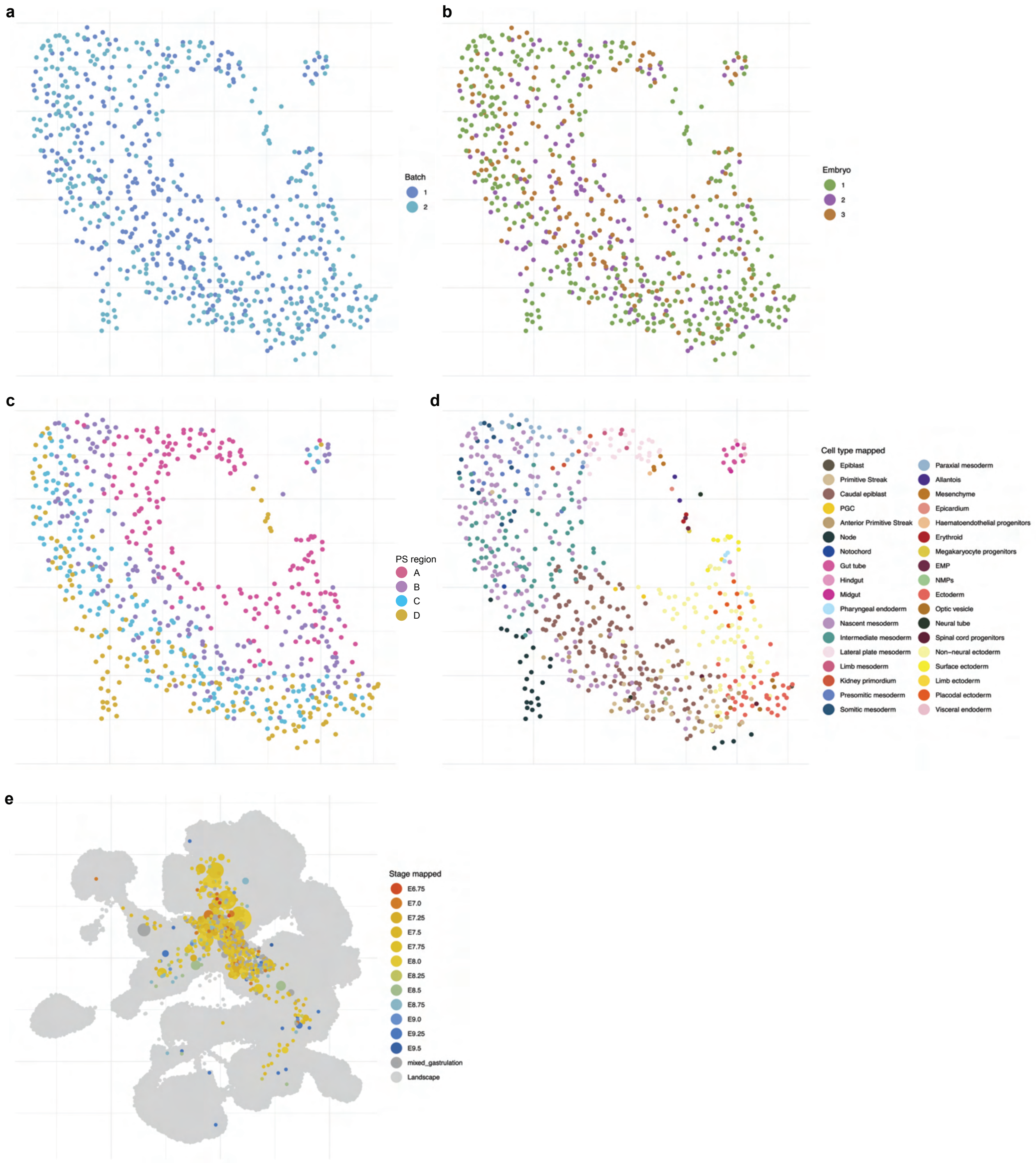

Fig. S14. Primitive streak dissections profiled with Smart-seq2.

UMAP layout of single cell transcriptomes from primitive streak dissections after batch correction (cells are coloured by batch). **b)** Three different embryos were profiled (cells are coloured by dissected embryos). **c)** Dissected portions of the Primitive Streak from Anterior to Posterior (cells are coloured by portions). **d)** Cell type annotations assigned to cells by transferring annotation from the whole atlas (cells are coloured by cell types). **e)** UMAP of primitive streak cells mapped onto the extended mouse atlas. Cells are coloured by the embryo stage corresponding to its closest neighbour in the atlas.

Development • Supplementary information

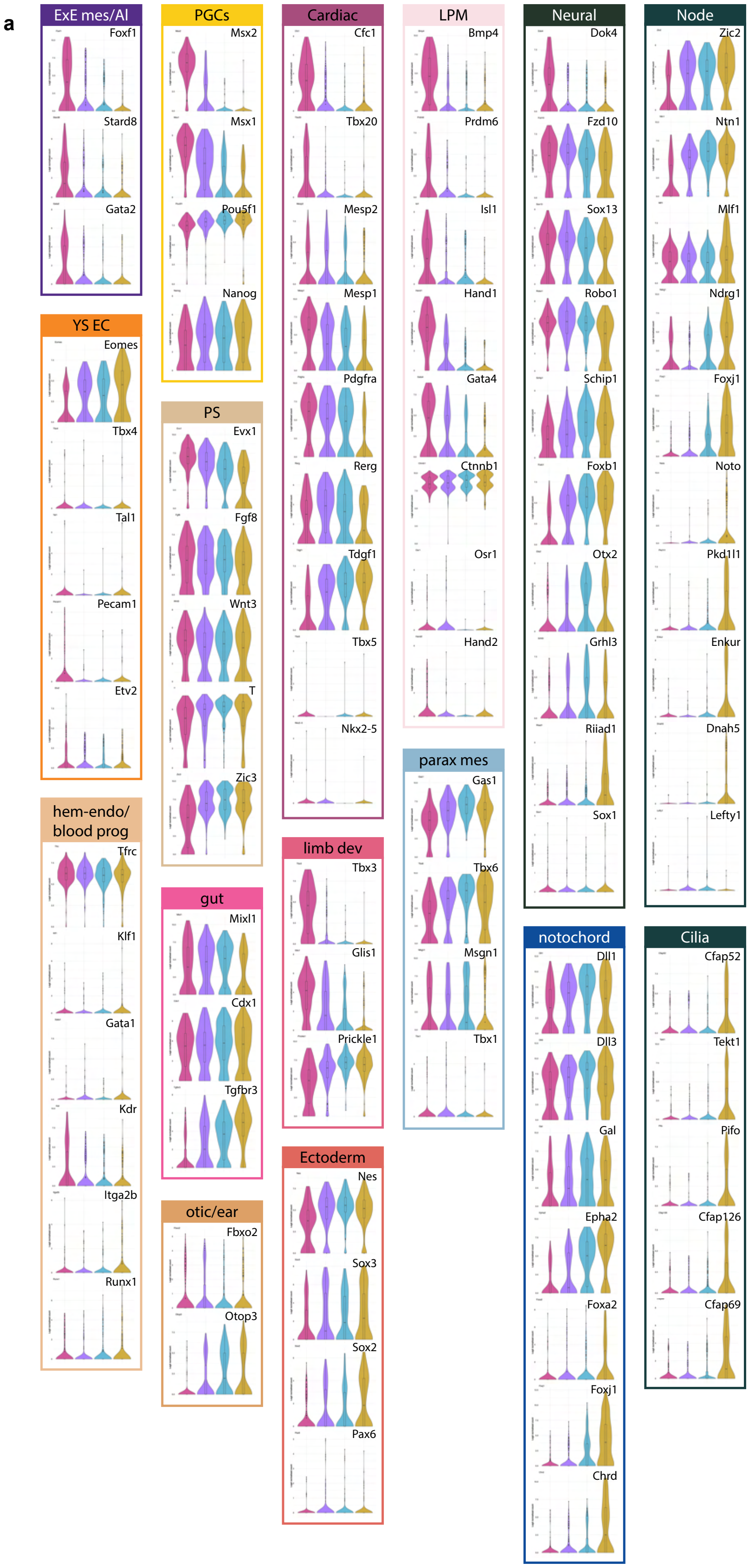
a
ExE mes/Al
Foxf1
Stard8
Gata2
YS EC
Eomes
Tbx4
Tal1
Pecam1
Etv2
hem-endo/ blood prog
Tfrc
Klf1
Gata1
Kdr
Itga2b
Runx1
PGCs
Msx2
Msx1
Pou5f1
Nanog
PS
Evx1
Fgf8
Wnt3
T
Zic3
gut
Mixl1
Cdx1
Tgfbr3
otic/ear
Fbxo2
Otop3
Cardiac
Cfc1
Tbx20
Mesp2
Mesp1
Pdgfra
Rerg
Tdgf1
Tbx5
Nkx2-5
limb dev
Tbx3
Glis1
Prickle1
Ectoderm
Nes
Sox3
Sox2
Pax6
LPM
Bmp4
Prdm6
Isl1
Hand1
Gata4
Ctnnb1
Osr1
Hand2
parax mes
Gas1
Tbx6
Msgn1
Tbx1
Neural
Dok4
Fzd10
Sox13
Robo1
Schip1
Foxb1
Otx2
Grhl3
Riiad1
Sox1
notochord
Dll1
Dll3
Gal
Epha2
Foxa2
Foxj1
Chrd
Node
Zic2
Ntn1
Mlf1
Ndrg1
Foxj1
Noto
Pkd1l1
Enkur
Dnah5
Lefty1
Cilia
Cfap52
Tekt1
Pifo
Cfap126
Cfap69

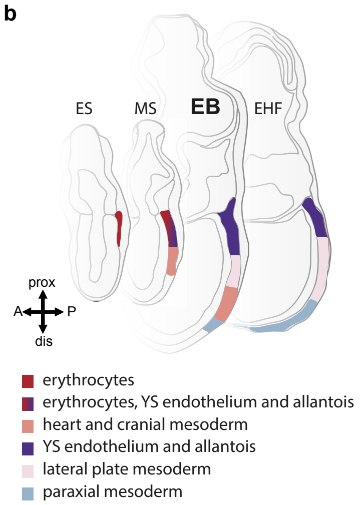
b
ES
MS
EB
EHF
prox
A
P
dis
erythrocytes
erythrocytes, YS endothelium and allantois
heart and cranial mesoderm
YS endothelium and allantois
lateral plate mesoderm
paraxial mesoderm

Fig. S15. Examples of cell type-specific gene signatures within primitive streak region A to D.

a) Violin plots of cell type-specific gene signatures within the 4 primitive streak regions are shown. Cell types are colour coded according to the UMAP legend (Fig.4b). **b)** Schematic representation of previously established primitive streak fates at different embryonic stages during gastrulation, adapted from Kinder et al., 2001.

Development • Supplementary information

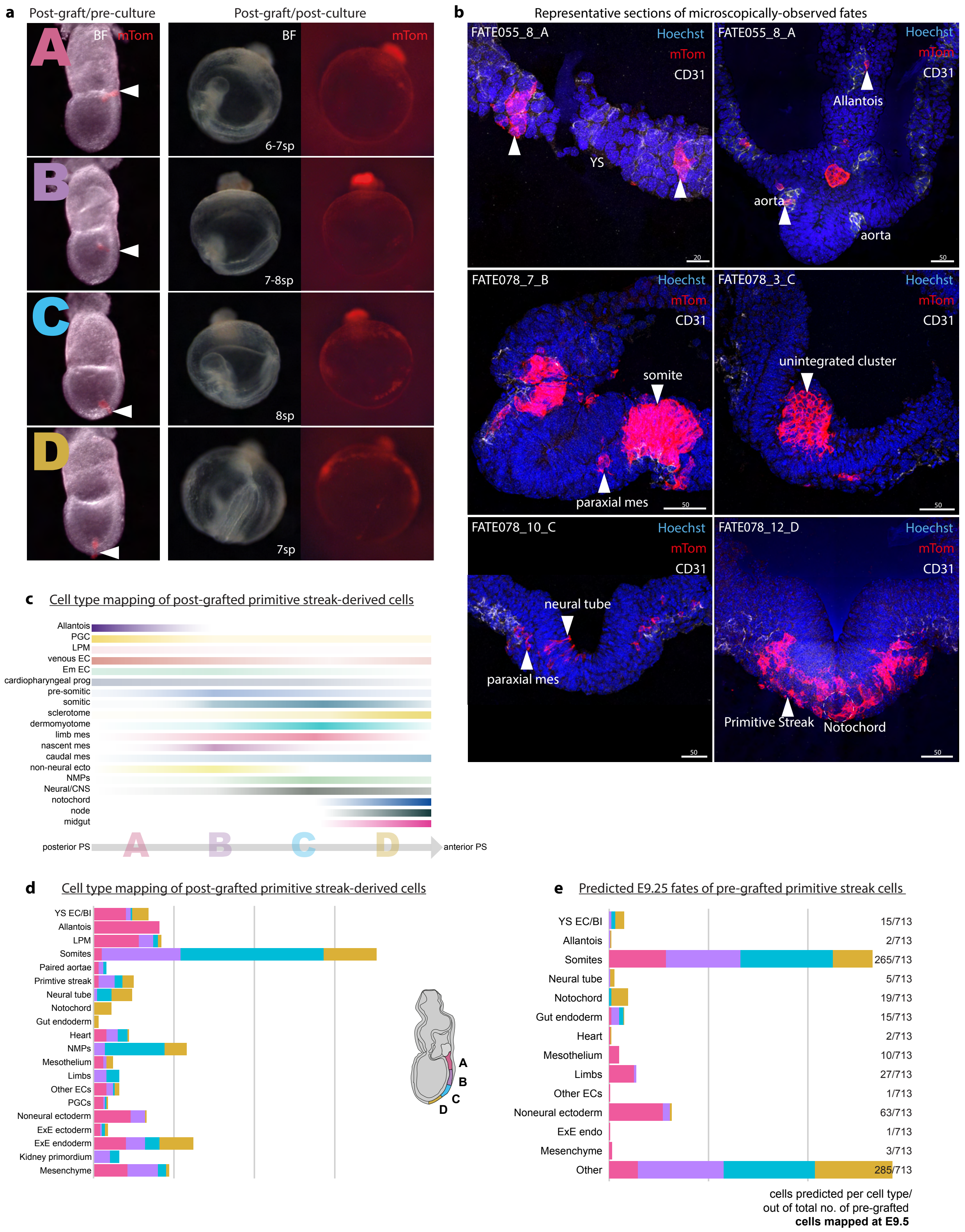
a
Post-graft/pre-culture
Post-graft/post-culture
A
B
C
D
BF
mTom
6-7sp
7-8sp
8sp
7sp
b
Representative sections of microscopically-observed fates
FATE055_8_A
Hoechst
mTom
CD31
YS
Allantois
aorta
aorta
FATE078_7_B
somite
paraxial mes
FATE078_3_C
unintegrated cluster
FATE078_10_C
neural tube
paraxial mes
FATE078_12_D
Primitive Streak
Notochord
c
Cell type mapping of post-grafted primitive streak-derived cells
Allantois
PGC
LPM
venous EC
Em EC
cardiopharyngeal prog
pre-somitic
somitic
sclerotome
dermomyotome
limb mes
nascent mes
caudal mes
non-neural ecto
NMPs
Neural/CNS
notochord
node
midgut
posterior PS
anterior PS
d
Cell type mapping of post-grafted primitive streak-derived cells
YS EC/Bl
Allantois
LPM
Somites
Paired aortae
Primtive streak
Neural tube
Notochord
Gut endoderm
Heart
NMPs
Mesothelium
Limbs
Other ECs
PGCs
Noneural ectoderm
ExE ectoderm
ExE endoderm
Kidney primordium
Mesenchyme
e
Predicted E9.25 fates of pre-grafted primitive streak cells
YS EC/Bl 15/713
Allantois 2/713
Somites 265/713
Neural tube 5/713
Notochord 19/713
Gut endoderm 15/713
Heart 2/713
Mesothelium 10/713
Limbs 27/713
Other ECs 1/713
Noneural ectoderm 63/713
ExE endo 1/713
Mesenchyme 3/713
Other 285/713
cells predicted per cell type/
out of total no. of pre-grafted
cells mapped at E9.5

Fig. S16. Grafting controls and full fate maps.

a) Wholemount images showing EB-stage wild type embryos orthotopically grafted with primitive streak regions A-D of mTom transgenic mouse embryos. Arrowheads point to the grafting site along the primitive streak axis with visible red cluster cells lodged in the primitive streak immediately after grafting and before embryo culture. After 24h culture, embryos developed around 7 somite pairs (Suppl.Table 1), had a beating heart and showed mTom contribution in their tissues. **b)** Representative immunofluorescent images of post-grafted/post-cultured embryo sections, where mTom contribution was assessed based on microscopic observations. **c)** Percentile representation of cell type mapping along the posterior-anterior axis of the primitive streak (from A to D, respectively) of post-grafted cells. The colour intensity indicates cell type abundance and resembles the gradient of cell type bias from specific primitive streak regions. **d)** Full fate map of transcriptionally observed fates based on the mapping of the post-grafted cells on the extended atlas. **e)** Full fate map of predicted fates of the pre-graft PS portion cells, determined with the Waddington-OT algorithm. Prediction was made including the full extended atlas, thus up to E9.25.

Table S1. Cell type markers across entire extended gastrulation atlas

Available for download at
https://journals.biologists.com/dev/article-lookup/doi/10.1242/dev.201867#supplementary-data

Table S2. Referencing genes markers for cell type annotation

Available for download at
https://journals.biologists.com/dev/article-lookup/doi/10.1242/dev.201867#supplementary-data

Table S3. Tradeseq Output: Genes that change along the YS Primitive, YS Definitive Blood and YS Endothelial Inferred Trajectories

Available for download at
https://journals.biologists.com/dev/article-lookup/doi/10.1242/dev.201867#supplementary-data

Table S4. Tradeseq Output: Genes that change along the Anterior/Medial Endothelial Inferred Trajectories with GO Terms for Clustered Genes

Available for download at
https://journals.biologists.com/dev/article-lookup/doi/10.1242/dev.201867#supplementary-data

Table S5. Tradeseq Output: Genes that change along the Posterior Endothelial Inferred Trajectories with GO Terms for Clustered Genes

Available for download at
https://journals.biologists.com/dev/article-lookup/doi/10.1242/dev.201867#supplementary-data

Table S6. Deeper characterization of endotome marker gene expression

Available for download at
https://journals.biologists.com/dev/article-lookup/doi/10.1242/dev.201867#supplementary-data

Table S7. CellComm Output: Ligand-Receptor Interactions in the YS Landscape and the Hematoendothelial Landscape

Available for download at
https://journals.biologists.com/dev/article-lookup/doi/10.1242/dev.201867#supplementary-data

Table S8. Fate contributions of orthotopically grafted primitive streak regions

Available for download at
https://journals.biologists.com/dev/article-lookup/doi/10.1242/dev.201867#supplementary-data
